# Accommodating informative dropout and death: a joint modelling approach for longitudinal and semicompeting risks data

**DOI:** 10.1111/rssc.12210

**Published:** 2017-01-30

**Authors:** Qiuju Li, Li Su

**Affiliations:** ^1^ Medical Research Council Biostatistics Unit Cambridge UK

**Keywords:** Joint models, Missing data, Shared parameter models, Survival analysis

## Abstract

Both dropout and death can truncate observation of a longitudinal outcome. Since extrapolation beyond death is often not appropriate, it is desirable to obtain the longitudinal outcome profile of a population given being alive. We propose a new likelihood‐based approach to accommodating informative dropout and death by jointly modelling the longitudinal outcome and semicompeting event times of dropout and death, with an important feature that the conditional longitudinal profile of being alive can be conveniently obtained in a closed form. We use proposed methods to estimate different longitudinal profiles of CD4 cell count for patients from the ‘HIV Epidemiology Research Study’.

## 1 Introduction

### 1.1 Dropout mixed with death in longitudinal studies

In long‐term longitudinal studies, both dropout (i.e. patients' withdrawal due to reasons other than death) and death can occur during the follow‐up, which truncates the observation of the longitudinal outcome of interest for a patient. For example, in a study of human immunodeficiency virus (HIV), CD4 cell count as the primary outcome of disease progression is scheduled to be repeatedly measured at planned follow‐up visits, but both patients' withdrawal and death can terminate the observation of CD4 cell counts for a patient during the study period. This ‘dropout mixed with death’ problem started to attract attention in the literature a decade ago, primarily driven by applications in studies of aging (Dufouil *et al*., 2004; Rajan and Leurgans, 2010). Depending on research aims, there are generally three classes of models that deal with both dropout and death (Kurland *et al*., 2009).

*Unconditional* models, such as random‐effects models fitted to the observed longitudinal data only, are appropriate if deaths are independent of the longitudinal outcome. They can also be used if deaths do not result in truncation because the longitudinal outcome is well defined after death (e.g. medical costs). In these models, the longitudinal outcome may be implicitly imputed beyond death and the targeted population is often termed an ‘immortal cohort’ (Dufouil *et al*., 2004). In other words, the inference is for a hypothetical population that is free of death during the study period. Using a multiple inverse probability weighting approach, Rajan and Leurgans (2010) also developed an unconditional model to account for both dropout and death (Harel and Demirtas, 2011).

In *fully conditional* models, separate regression models can be specified for strata that are defined by the time of death (Ribaudo *et al*., 2000; Pauler *et al*., 2003), similarly to pattern–mixture models in the missing data literature, or the time of death can be included as a covariate. The ‘principal stratification’ method, which is used to estimate the causal effect of a treatment, is also a type of fully conditional models, where strata are based on the counterfactual survival times under both treatment groups (Frangakis and Rubin, 2002; Rubin, 2006; Shardell *et al*., 2015). Note that fully conditional models require exact times of death to be conditioned on. Therefore censoring of survival information is not accommodated in these models.

*Partly conditional* models focus on the distribution of the longitudinal outcome conditionally on being alive at a specific time point; therefore the targeted population is a dynamic cohort of survivors or a ‘mortal cohort’. So far methods for fitting partly conditional models have been based on weighted estimating equations (Dufouil *et al*., 2004; Kurland and Heagerty, 2005; Shardell and Miller, 2008; Shardell *et al*., 2010), where most of them did not focus on informative dropout, and censoring of survival information other than dropout as well as intermittent missingness were not allowed.

In contrast, to the best of our knowledge, likelihood‐based approaches that have the potential to address these issues (informative dropout, censoring of survival information and intermittent missingness) have not been developed for partly conditional models.

### 1.2 Joint modelling with longitudinal and semicompeting risks data

In this paper, we propose a new likelihood‐based approach to dealing with both informative dropout and death. Because dropout can be censored by death but the reverse is not true if times of death are available from public records after dropout, we treat dropout and death as semicompeting risks. Starting from an unconditional model for the immortal cohort, we build a joint model (JM) for the longitudinal outcome and the semicompeting risks by using the general framework of JMs of longitudinal and time‐to‐event data. The associations between the longitudinal outcome and the two semicompeting risks are characterized by shared random effects.

Specifically, building on the JMs that were proposed in Barrett *et al*. (2015), we assume a linear mixed model (LMM) for the longitudinal outcome in the immortal cohort. As the exact time of dropout is often unavailable in practice, the timescale that we use for the time of dropout is the times of scheduled visits for the longitudinal outcome and hence it is discrete. The exact time of death is usually available from public records but discretized such that it has the same timescale as the time of dropout. We assume two separate probit models for the discrete time hazards of dropout and death. Linear combinations of the random effects in the submodel for the longitudinal outcome are included in the submodels for dropout and death to characterize the associations between the evolutions of the three outcomes over time.

Compared with existing moment‐based methods for *partly conditional* models (Kurland and Heagerty, 2005), our approach has the following advantages.

- Informative dropout and outcome‐related death can be handled, whereas existing methods have focused on ignorable dropout.
- Existing methods have treated the dropout and death as competing risks and only allowed death to be censored by dropout, whereas our approach treats them as semicompeting risks and allows independent censoring of death before dropout.
- Intermittent missing data are allowed within the likelihood‐based framework; in particular, we assume that the probability of intermittent missingness is independent of the intermittent missing longitudinal outcome given observed data. Therefore, explicit modelling of the indicator of intermittent missingness is not required.
- Model assessment for the fit to the observed data is straightforward within the likelihood‐based framework. For example, with Bayesian estimation, we can use posterior predictive checks based on replicated observed data as recommended in Daniels *et al*. (2012).

Compared with *unconditional* models (Rajan and Leurgans, 2010; Harel and Demirtas, 2011), in addition to the inference for the unconditional model parameterized for the longitudinal outcome, our approach can conveniently provide the conditional longitudinal outcome profile given being alive (i.e. inferences for the mortal cohort) because the random effects conditional on being alive at some time point follow a multivariate skew normal distribution. Compared with *fully conditional* models (Ribaudo *et al*., 2000; Pauler *et al*., 2003), exact death time information is not required in our approach; and our approach can conveniently provide inferences for both the unconditional and the partly conditional longitudinal profiles, whereas fully conditional models require numerical integration over the death time distribution to provide the conditional longitudinal profile given being alive.

Our methods also contribute to the literature on joint modelling of longitudinal and time‐to‐event data as it appears that semicompeting risks data have not been addressed, although there are various models to deal with competing risks data in the joint modelling framework (Elashoff *et al*., 2007, 2008; Williamson *et al*., 2008; Hu *et al*., 2009; Proust‐Lima *et al*., 2016).

For estimation, both maximum likelihood and Bayesian approaches can be used. Maximum likelihood estimates (MLEs) can be obtained by maximizing the marginal likelihood after integrating over the random effects. Limitations of this approach are that the computation can be very intensive and we need to sample from the asymptotic distribution of the MLE to obtain confidence intervals for the estimated longitudinal profiles conditionally on being alive. In the analysis that is presented in Section 4, we also discover some computational issues related to calculating high dimensional multivariate normal probabilities in the maximum likelihood estimation. Details will be discussed in Sections 4 and 6. In contrast, the Bayesian approach does not require the integration of random effects and can directly provide the posterior inferences for longitudinal profiles conditionally on being alive since they are functions of the model parameters. In this paper, we implement both estimation approaches in our application in Section 4.

### 1.3 ‘HIV Epidemiology Research Study’

This work is motivated by data from the ‘HIV Epidemiology Research Study’ (HERS). The HERS was a longitudinal study of 1310 women with, or at high risk for, HIV infection from 1993 to 2000 (Smith *et al*., 2003). During the study 12 visits were scheduled, where a variety of clinical, behavioural and sociological outcomes were recorded approximately every 6 months. We shall focus on the 850 women who were HIV positive and had a CD4 cell count measurement at enrolment.

**Figure 1:**
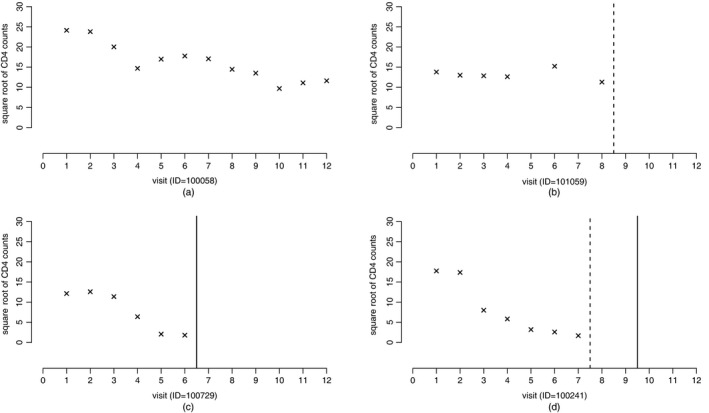
Observed data of four HERS patients that represent four scenarios of dropout and death in the cohort (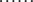, observed dropout times; 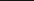, observed death times; ×, square root of the observed CD4 cell count data for the patients): (a) complete data; (b) dropout; (c) death; (d) dropout and death

There were 106 HIV‐related deaths during the study follow‐up. In addition, censoring by dropout also occurred, which was possibly related to the disease progression characterized by the CD4 cell count outcome, as suggested by previous analyses of these data (Hogan *et al*., 2004). In other words, the dropout is probably informative. Fig. 1 shows the data from four HERS patients, and they represent the four scenarios of dropout and HIV‐related death times in the cohort, together with their observed CD4 cell count data over time (a square root transformation is used to reduce the right skewness in these data). Previous analyses of the HERS data (Hogan *et al*., 2004; Daniels and Hogan, 2008) did not distinguish between censoring by dropout and death. As the CD4 cell count outcome is not appropriate beyond death, the mortal cohort inference for these data is certainly of interest. In other words, it is desirable to impute missing CD4 cell counts after dropout but not to impute CD4 cell counts beyond death. Hence our aim is to obtain the mortal cohort inference for the CD4 cell count outcome while dealing with both informative dropout and death, which motivates our new likelihood‐based approach.

The rest of the paper is organized as follows. In Section 2, we introduce our JM. Estimation and inference are described in Section 3. In Section 4, we apply the proposed methods to the HERS data and demonstrate the differences between the inferences for mortal and immortal cohorts. A brief simulation study is performed in Section 5 to examine the finite sample performance of the methods proposed. We conclude with a discussion in Section 6.

The programs that were used to analyse the data can be obtained from

http://wileyonlinelibrary.com/journal/rss-datasets

## 2 Joint model

Suppose that *N* independent patients are followed up over time. For the *i*th (*i*=1,…,*N*) patient, longitudinal measurements **Y**_*i*_=(*Y*_*i*1_,…,*Y*_*iM*_)^T^ are scheduled to be taken over time in T=[0,T], where *T* is the total length of scheduled follow‐up in the study. However, patients can withdraw from the study (drop out) or die during the follow‐up, which can both terminate the observation of the longitudinal outcome **Y**_*i*_.

Let *D*_*i*_ and *S*_*i*_ denote the time of dropout and the time of death for the *i*th patient. Information about the exact time of dropout is often not available in practice. Therefore *D*_*i*_ is usually assumed to be the number of the last follow‐up visit and hence is treated as discrete. However, exact survival information for the patients can often be obtained from public records during the study follow‐up, even after the patients drop out. Therefore we treat dropout and death as semicompeting risks since death can terminate the dropout process but the reverse is not true.

For death time *S*_*i*_, instead of using the continuous timescale T=[0,T], we assume a discrete timescale S={1,2,…,M}. However, it is assumed that there is a surjection *s*(*t*) from T to S; for example, S might result from a partition of T. Then S is considered to be a series of time intervals such that each of them contains a scheduled visit for **Y**_*i*_. Further, during the study both *D*_*i*_ and *S*_*i*_ can be censored at *C*_*i*_, the censoring time. We assume that *C*_*i*_ is independent of *D*_*i*_ and *S*_*i*_. For example, *C*_*i*_=*M* if the patient completes the study and therefore both *D*_*i*_ and *S*_*i*_ are administratively censored. The observed time of dropout is $D_{i}^{*}=\min\left(D_{i},S_{i},C_{i}\right)$ and the indicator for dropout occurrence is $\delta_{i}^{D}=I\left(D_{i}⩽C_{i},D_{i}⩽S_{i}\right)$. The observed time of death is $S_{i}^{*}=\min\left(S_{i},C_{i}\right)$, and the indicator for death occurrence is $\delta_{i}^{S}=I\left(S_{i}⩽C_{i}\right)$. By definition $D_{i}^{*}⩽S_{i}^{*}$ and the observed longitudinal measurements after truncation by dropout and death are $Y_{i}^{o}={\left(Y_{i1},\ldots ,Y_{in_{i}}\right)}^{T}$ and $n_{i}⩽D_{i}^{*}$.

We assume that the associations between the longitudinal outcome and the two semicompeting risks are characterized by random effects **b**_*i*_ and covariates. In our motivating application, this is a reasonable assumption because the longitudinal outcome (e.g. CD4 cell count) characterizes HIV disease progression, and HIV disease progression can influence both the dropout and survival. Given **b**_*i*_ and covariates, the *complete* longitudinal outcome **Y**_*i*_, the dropout time *D*_*i*_ and the death time *S*_*i*_ are assumed to be independent.

### 2.1 Longitudinal submodel

We assume the following model for *Y*_*ij*_ (*j*=1,…,*M*):

(1) $$Y_{ij}=x_{ij}^{T}\beta +z_{ij}^{T}b_{i}+\epsilon_{ij},$$

where ***β*** is a *p*×1 vector of regression coefficients associated with exogenous covariates **x**_*ij*_ (fixed effects), **b**_*i*_ is a *q*×1 vector of random effects that are associated with covariates **z**_*ij*_, *ε*_*ij*_ is the measurement error that is independent of covariates **x**_*ij*_ and **z**_*ij*_ and (*ε*_*i*1_,…,*ε*_*iM*_)∼*N*(**0**,*V*_*i*_), where *V*_*i*_ can be any positive definite covariance matrix. In the HERS application, we assume that $V_{i}=\sigma_{\epsilon }^{2}I_{M\times M}$ (*I* is an identity matrix) to account for measurement errors only and the random effects are used to capture the serial correlations over time for longitudinal data. In practice, other parametric models such as auto‐regressive models can be used for *V*_*i*_ to characterize the remaining serial correlations. **b**_*i*_ are assumed normally distributed with mean 0 and covariance matrix Σ and are independent of *ε*_*ij*_ and covariates **x**_*ij*_ and **z**_*ij*_. Note that model (1) is an unconditional model (i.e. unconditional on the time of death given the covariates) for characterizing the longitudinal outcome in the immortal cohort.

### 2.2 Submodel for semicompeting risks

Following Barrett *et al*. (2015), we assume a probit model for the discrete time hazard of dropout $\lambda_{D,\;ij}=P\left(D_{i}=j|D_{i}⩾j,b_{i},x_{D,\;ij},W_{D,\;ij}\right)$ at the *j*th visit:

(2) $$\lambda_{D,\;ij}=1-\Phi \left\{x_{D,\;ij}^{T}\alpha^{D}+{\left(W_{D,\;ij}b_{i}\right)}^{T}\gamma_{j}^{D}\right\},$$

where Φ(·) is the standard normal cumulative distribution function, **x**_*D*,*ij*_ is a *p* ^*D*^×1 vector of covariates (possibly time varying) with regression coefficients ***α*** ^*D*^,*W*_*D*,*ij*_ is a matrix for constructing a *q* ^*D*^×1 vector of linear combinations of **b**_*i*_, *W*_*D*,*ij*_ **b**_*i*_ (for example, in the HERS application, we have *W*_*D*,*ij*_=*I* and *q* ^*D*^=2), and $\gamma_{j}^{D}$ is an association parameter vector that relates the longitudinal outcome and the dropout time via the random effects **b**_*i*_. If $\gamma_{j}^{D}=0$ then the dropout is ignorable given the observed data.

We also assume a probit model for the discrete time hazard of death $\lambda_{S,\;ij}=P\left(S_{i}=j|S_{i}⩾j,b_{i},x_{S,\;ij},W_{S,\;ij}\right)$ at the *j*th visit:

(3) $$\lambda_{S,\;ij}=1-\Phi \left\{x_{S,\;ij}^{T}\alpha^{S}+{\left(W_{S,\;ij}b_{i}\right)}^{T}\gamma_{j}^{S}\right\},$$

where **x**_*S*,*ij*_ is a *p* ^*S*^×1 vector of covariates with regression coefficients ***α*** ^*S*^. *W*_*S*,*ij*_ **b**_*i*_ is a *q* ^*S*^×1 vector of linear combinations of **b**_*i*_ and $\gamma_{j}^{S}$ is an association parameter vector that relates the longitudinal outcome and the time of death. If $\gamma_{j}^{S}=0$, then the time of death is independent of the longitudinal outcome and censoring by death is non‐informative given the observed data.

## 3 Estimation and inference

### 3.1 Likelihood

In this section, we derive the complete‐data likelihood conditioning on the random effects and for simplicity of presentation we suppress the conditioning on the covariates **x**_*ij*_, **z**_*ij*_, **x**_*D*,*ij*_, *W*_*D*,*ij*_, **x**_*S*,*ij*_ and *W*_*S*,*ij*_. The observed data for the outcomes are $\left\{Y_{i}^{o},D_{i}^{*}=d,\delta_{i}^{D},S_{i}^{*}=s,\delta_{i}^{S}\right\}$ (*i*=1,…,*N*), and the complete‐data likelihood from the *i*th patient is

(4) $$L_{i}\left(\theta |Y_{i}^{o},D_{i}^{*}=d,\delta_{i}^{D},S_{i}^{*}=s,\delta_{i}^{S},b_{i}\right)=f\left(Y_{i}^{o}|b_{i};\theta \right)f\left(d,\delta_{i}^{D}|b_{i};\theta \right)f\left(s,\delta_{i}^{S}|b_{i};\theta \right)f\left(b_{i};\theta \right),$$

where ***θ*** denotes all unknown parameters in the JM. Let $X_{i}={\left(x_{i1},\ldots ,x_{in_{i}}\right)}^{T}$ and $Z_{i}={\left(z_{i1},\ldots ,z_{in_{i}}\right)}^{T}$. The likelihood from the longitudinal part given the random effects is

$$f\left(Y_{i}^{o}|b_{i};\theta \right)=\exp\{-\log\left(2\pi \right)n_{i}/2-\log(|V_{i}\left|\right)/2-{\left(Y_{i}^{o}-\mu_{i}\right)}^{T}V_{i}^{-1}\left(Y_{i}^{o}-\mu_{i}\right)/2\},$$

where ***μ***_*i*_=*X*_*i*_ ***β***+*Z*_*i*_ **b**_*i*_ and for the HERS data we assume that $V_{i}=\sigma_{\epsilon }^{2}I_{n_{i}\times n_{i}}$.

The likelihood from the dropout part given the random effects is

(5) $$\begin{array}{cc} f(d,\delta_{i}^{D}|b_{i};\theta ) & =\left[\prod_{j=1}^{d-1}\Phi \left\{x_{D,\;ij}^{T}\alpha^{D}+{\left(W_{D,\;ij}b_{i}\right)}^{T}\gamma_{j}^{D}\right\}\right]{\left[\Phi \left\{x_{D,id}^{T}\alpha^{D}+{\left(W_{D,\;id}b_{i}\right)}^{T}\gamma_{d}^{D}\right\}\right]}^{1-\delta_{i}^{D}}\\ & \times {\left[1-\Phi \left\{x_{D,\;id}^{T}\alpha^{D}+{\left(W_{D,\;id}b_{i}\right)}^{T}\gamma_{d}^{D}\right\}\right]}^{\delta_{i}^{D}}. \end{array}$$

The likelihood from the survival part given the random effects is

(6) $$\begin{array}{cc} f(s,\delta_{i}^{S}|b_{i};\theta ) & =\left[\prod_{j=1}^{s-1}\Phi \left\{x_{S,\;ij}^{T}\alpha^{S}+{\left(W_{S,ij}b_{i}\right)}^{T}\gamma_{j}^{S}\right\}\right]{\left[\Phi \left\{x_{S,is}^{T}\alpha^{S}+{\left(W_{S,\;is}b_{i}\right)}^{T}\gamma_{s}^{S}\right\}\right]}^{1-\delta_{i}^{S}}\\ & \times {\left[1-\Phi \left\{x_{S,\;is}^{T}\alpha^{S}+{\left(W_{S,\;is}b_{i}\right)}^{T}\gamma_{s}^{S}\right\}\right]}^{\delta_{i}^{S}}. \end{array}$$

The density *f*(**b**_*i*_;***θ***) is that of the multivariate normal distribution *N*(**0**,Σ).

### 3.2 Cholesky decomposition for covariance matrix of random effects

Let *b*_*ik*_ be the *k*th element of the random effects **b**_*i*_ and *k*=1,…,*q*. We use a modified Cholesky decomposition to parameterize the random‐effects covariance matrix Σ to guarantee its positive definiteness (Daniels and Zhao, 2003). Recall that the random effects **b**_*i*_∼*N*(**0**,Σ). Following Daniels and Zhao (2003), let ${\tilde{b}}_{ik}$ (*k*=1,…,*q*) be the linear least squares predictor of *b*_*ik*_ based on its predecessors *b*_*i*(*k*−1)_,…,*b*_*i*1_, and let $e_{ik}=b_{ik}-{\tilde{b}}_{ik}$ be the prediction error with variance $\sigma_{k}^{2}=\text{var}\left(e_{ik}\right)$, i.e.

(7) $$b_{ik}=\sum_{l=1}^{k-1}\lambda_{kl}b_{il}+e_{ik},$$

where *λ*_*kl*_ are referred to as generalized auto‐regressive parameters and $\sigma_{k}^{2}$ as innovation variances. The special Cholesky decomposition of Σ is defined as *L*Σ*L* ^T^=*G*, where *L* is the lower unit triangular matrix with −*λ*_*kl*_ as its (*k*,*l*)th entry and $G=\text{diag}(\sigma_{1}^{2},\ldots ,\sigma_{q}^{2})$. The only constraint that is needed for Σ to be positive definite is that $\sigma_{k}^{2}>0$ for all *k*.

In the HERS analysis we assume a simple case of **b**_*i*_=(*b*_*i*1_,*b*_*i*2_)^T^, where *b*_*i*1_ is a random intercept and *b*_*i*2_ is a random slope. Then equation (7) can be written in two parts:

(8) $$\begin{array}{cc} & b_{i1}=e_{i1},\\ & b_{i2}=\lambda_{21}b_{i1}+e_{i2}, \end{array}$$

where $\text{var}\left(e_{ik}\right)=\sigma_{k}^{2}$, *k*=1,2. The first equation corresponds to the marginal distribution of the random intercepts, and the second equation describes the conditional distribution of random slopes given random intercepts. Thus the covariance matrix Σ is

$$\Sigma =\left(\begin{array}{cc} \sigma_{1}^{2} & \lambda_{21}\sigma_{1}^{2}\\ \lambda_{21}\sigma_{1}^{2} & \lambda_{21}^{2}\sigma_{1}^{2}+\sigma_{2}^{2} \end{array}\right)\;\;.$$

### 3.3 Estimation

The random effects in the likelihood (4) can be integrated out and the resulting marginal likelihood can be written in a closed form with well‐defined functions (Arnold, 2009; Barrett *et al*., 2015). Details can be found in the on‐line supplementary materials. It is straightforward to use the maximum likelihood approach for estimation, and the Hessian matrix can be used to approximate the standard errors. Specifically, obtaining the marginal likelihood involves calculating the cumulative probabilities of multivariate normal distributions, which can be implemented in R with the mnormt and mvtnorm packages.

The maximum‐likelihood‐based approach, however, can be very challenging and time demanding when the multivariate normal probability calculation is of high dimension and the number of parameters is large. For example, there were 12 scheduled visits in the HERS and as a result the dimension of multivariate normal probability calculation can be up to 24. In such a case, the Monte‐Carlo‐based approach (in the mvtnorm package) approximates the marginal likelihood, which introduces Monte Carlo error and consequently causes convergence problems and problems with the numerical evaluation of the Hessian matrix. More details will be provided in Section 4 for the HERS analysis. In practice, given the necessary model building and selection process, it is therefore more efficient to use a Bayesian approach when the multivariate normal probability is of high dimension. We shall give details of the prior specification and posterior inference in the HERS analysis. Computation for the Bayesian approach is implemented in the WinBUGS package (Spiegelhalter *et al*., 2003). For the HERS data analysis, we demonstrate both the maximum likelihood and the Bayesian approaches.

### 3.4 Marginal mean profile conditional on being alive

Recall that the population mean profile in model (1) is $E\left(Y_{ij}|x_{ij},z_{ij}\right)=x_{ij}^{T}\beta$. To obtain the conditional mean profile given being alive, we can compute

(9) $$E\left(Y_{ij}|S_{i}⩾j,x_{ij},z_{ij}\right)=x_{ij}^{T}\beta +z_{ij}^{T}E\left(b_{i}|S_{i}⩾j,x_{ij},z_{ij}\right).$$

Although we assume that **b**_*i*_ and **x**_*ij*_ and **z**_*ij*_ are independent *a priori*, after conditioning on *S*_*i*_⩾*j*, they are no longer independent and $E(b_{i}|S_{i}⩾j,x_{ij},z_{ij})$ is a function of **x**_*ij*_ and **z**_*ij*_.

It is easy to show that the conditional distribution of **b**_*i*_ given *S*_*i*_⩾*j* and the covariates is a multivariate skew normal distribution. Therefore we can calculate $E(b_{i}|S_{i}⩾j,x_{ij},z_{ij})$ as a function of specified parameters and covariates. Details are given in the on‐line supplementary materials.

## 4 Unconditional and conditional longitudinal profiles of CD4 cell counts in the ‘HIV Epidemiology Research Study’

In this section, we use the proposed methods to estimate the unconditional and conditional longitudinal profiles of CD4 cell counts as a function of baseline covariates (HIV viral load, HIV symptom severity and antiviral treatment indicator) from the HERS data that were introduced in Section 1 (Hogan *et al*., 2004). Of the 850 women who were HIV positive and had CD4 cell count data at baseline, we exclude 23 women from the analysis because their baseline covariate measurements were missing.

Attrition in the HERS is substantial, like in many other long‐term follow‐up studies. Table 1 shows that more than half of the women did not complete the study because of either early dropout or HIV‐related death. In particular, 78 women dropped out of the study before dying with HIV‐related reasons. Moreover, previous analyses of these data suggested that it is quite plausible that the dropout was closely related to the missing CD4 cell counts, i.e. the unobserved CD4 cell counts among those who dropped out are systematically lower than those who continued follow‐up, even after adjusting for covariates and observed CD4 cell counts (Hogan *et al*., 2004). We need to deal with this informative dropout in our analysis. Hogan *et al*. (2004) have examined the unconditional profile of CD4 cell counts and related factors, but dropout and HIV‐related death are not distinguished in their pattern–mixture model approach. In our analysis, we shall also investigate the conditional profile of CD4 cell counts given being alive, which might provide insights to clinical questions that were not addressed in previous analyses.

**Table 1:** Number of patients with different semicompeting risks data of dropout and HIV‐related death in the HERS

*Indicators for dropout or*	*Number of patients*
*HIV‐related death*	
$(\delta_{i}^{D},\delta_{i}^{S})=$ (0, 0)	374
$(\delta_{i}^{D},\delta_{i}^{S})=$ (1, 0)	352
$(\delta_{i}^{D},\delta_{i}^{S})=$ (0, 1)	23
$(\delta_{i}^{D},\delta_{i}^{S})=$ (1, 1)	78

For those women who actually finished 12 scheduled visits, their dropout and HIV‐related times of death are treated as administratively censored at the time of this final visit. The maximum follow‐up time was 2093 days in the HERS data, and we partition the follow‐up period into 12 intervals to determine the observed discretized time of death $S_{i}^{*}$ and $\delta_{i}^{S}$. Except for the first interval which is 3 months from enrolment, the remaining 11 intervals are equally spaced for 6 months such that each interval contains one scheduled CD4 cell count measurement. During the follow‐up, 579 (7.6%) CD4 cell count measurements were intermittently missing before the patients' dropout, death or the end of the study. We assume that this intermittent missingness is ignorable, i.e. the probability of intermittent missingness is assumed to be independent of the unobserved longitudinal outcome, given the observed data. Therefore, no additional model for the indicators of intermittent missingness is specified.

### 4.1 Fitted models

Following the previous analysis of the HERS data (Hogan *et al*., 2004), we assume the unconditional model for the longitudinal measurements of CD4 cell count as follows:

(10) $$Y_{ij}=x_{ij}^{T}\beta +b_{i1}+b_{i2}j+\epsilon_{ij},$$

where *Y*_*ij*_ is the square root of the CD4 cell count at the *j*th visit and **x**_*ij*_ is the vector of corresponding covariates, which include the visit number *j* (time), indicator variables for HIV viral load group (0,500], (500,5000], (5000,30000] (copies per millilitre) at baseline, an indicator of antiretroviral therapy at baseline, HIV symptomatology (presence of HIV‐related symptoms on a scale from 0 to 5) at baseline and the interactions between time and these baseline covariates. *b*_*i*1_ and *b*_*i*2_ are a random intercept and slope respectively, and they follow the multivariate normal distribution with mean 0 and covariance Σ, as parameterized in expression (8).

On the basis of some preliminary investigations and the findings in Smith *et al*. (2003), we assume the following models for the dropout and death times:

$$\begin{array}{cc} & \text{Pr}\left(D_{i}=j|D_{i}⩾j,x_{D,\;ij},b_{i1},b_{i2}\right)=1-\Phi \left(x_{D,\;ij}^{T}\alpha^{D}+\gamma_{1}^{D}b_{i1}+\gamma_{2}^{D}b_{i2}\right),\\ & \text{Pr}\left(S_{i}=j|S_{i}⩾j,x_{S,\;ij},b_{i1},b_{i2}\right)=1-\Phi \left(x_{S,\;ij}^{T}\alpha^{S}+\gamma_{1}^{S}b_{i1}+\gamma_{2}^{S}b_{i2}\right), \end{array}$$

where the covariate vectors **x**_*D*,*ij*_ and **x**_*S*,*ij*_ both include indicators of baseline HIV viral load groups, HIV symptomatology at baseline, an indicator of antiretroviral therapy at baseline and *j*/12 and (*j*/12)^2^, to account for the change in the discrete time hazards over time. More details can be found in Table 2.

**Table 2:** Results for the dropout and HIV‐related death parts of the JM analysis of the HERS data^a^

	*Results of Bayesian analysis*			*Results of MLE analysis*		
	*Mean*	*2.5%*	*97.5%*	*Estimate*	*2.5%*	*97.5%*
*Dropout*						
Intercept	1.052	0.849	1.274	1.033	0.899	1.149
*j*/12	1.190	0.380	1.912	1.058	0.572	1.347
(*j*/12)^2^	−1.733	−2.405	−1.007	−1.746	−1.931	−1.545
Baseline HIV viral load (copies ml^−1^)						
0–500	0.733	0.523	0.934	0.733	0.595	0.886
500–5000	0.650	0.464	0.831	0.648	0.537	0.776
5000–30000	0.269	0.064	0.469	0.271	0.137	0.422
>30000	Reference			Reference		
Baseline HIV symptoms	−0.011	−0.063	0.044	−0.011	−0.072	0.049
Antiretroviral therapy at baseline	−0.043	−0.154	0.073	−0.042	−0.164	0.082
$\gamma_{1}^{D}$	0.029	0.018	0.039	0.028	0.016	0.041
$\gamma_{2}^{D}$	0.443	0.349	0.537	0.442	0.339	0.546
*HIV‐related death*						
Intercept	3.472	2.842	4.161	3.350	2.947	3.702
*j*/12	−4.272	−6.106	−2.469	−4.783	−5.793	−4.028
(*j*/12)^2^	2.931	1.415	4.449	2.715	2.210	3.271
Baseline HIV viral load (copies ml^−1^)						
0–500	2.032	1.427	2.751	1.992	1.463	2.781
500–5000	1.194	0.811	1.619	1.194	0.901	1.501
5000–30000	0.539	0.149	0.938	0.561	0.280	0.882
>30000	Reference			Reference		
Baseline HIV symptoms	−0.121	−0.245	0.000	−0.126	−0.256	0.003
Antiretroviral therapy at baseline	−0.516	−0.789	−0.249	−0.538	−0.807	−0.267
$\gamma_{1}^{S}$	0.128	0.098	0.162	0.131	0.100	0.162
$\gamma_{2}^{S}$	1.192	0.911	1.547	1.174	0.981	1.407

a For results from the Bayesian approach, we present the posterior mean and 95% credible intervals. For maximum profile likelihood estimation results, we provide point estimates and 95% confidence intervals.

We use both Bayesian and maximum likelihood approaches described in Section 3 for estimation. For the Bayesian approach, we assign independent normal priors *N*(0,100) to ***β*** and the parameter *λ*_21_ in Σ. For parameters in the dropout and death models, we assign weakly informative *N*(0,4) priors to ***α*** ^*D*^, $\gamma_{1}^{D}$ and $\gamma_{2}^{D}$, ***α*** ^*S*^, and $\gamma_{1}^{S}$ and $\gamma_{2}^{S}$ because these models are specified at the probit scale. For variance component parameters, we assign the prior $\sigma_{\epsilon }^{2}∼\text{inverse‐gamma}\left(0.001,0.001\right)$ and $\sigma_{k}^{2}∼\text{inverse‐gamma}\left(0.01,1\right)$ (*k*=1,2) for Σ. We run three Markov chain Monte Carlo chains with diverse initial values and assess convergence within a 5000‐iteration burn‐in period by using history plots and Gelman and Rubin convergence statistics provided by the WinBUGS package. After convergence, pooled posterior samples of size 45000 are used for model inference.

For maximum likelihood estimation, we encountered some numerical challenges in analysing the HERS data. The R function optim is used to obtain the MLEs numerically. Owing to the sample size (i.e. 827) and the large number of the model parameters (i.e. 36) as well as the dimension of multivariate normal probability calculation (up to 24), it is computationally intensive when it comes to the evaluation of the log‐likelihood function and the iteration of searching for the MLE, even though good initial values are provided from the results of the Bayesian approach. Furthermore, it is impossible to reach a sufficiently small convergence tolerance (e.g. 10^−6^), because of the Monte Carlo error that is introduced by the R package mvtnorm when evaluating the log‐likelihood function. For the HERS data, we stop the program at 2000 iterations, where we find that the log‐likelihood function values cannot be further improved with more iterations. Obtaining the Hessian matrix can also be a challenge when it needs to be approximated numerically, because a proper step value for the magnitude of gradient evaluation must be chosen not only to capture the characteristics of the log‐likelihood function but also to overcome the noise that is caused by the Monte Carlo error. Different choices of this step value can be tried out at this stage. However, this is to some extent arbitrary and the positive definiteness of the approximated Hessian matrix cannot be guaranteed. Therefore, we choose to use the profile likelihood approach for estimating the regression coefficient parameters and constructing the 95% confidence intervals. For each regression coefficient a profile likelihood function is evaluated at each of 10 grid point values of this parameter, by maximizing the likelihood with respect to the rest of the parameters. Because of the computational burden, we smooth the profile likelihood function to obtain the point estimate and 95% confidence interval based on the likelihood ratio test. We shall further discuss these computational issues in Section 6.

For comparison, we also present the results from fitting the LMM (as in equation (10)) to the HERS data without addressing the informative dropout and HIV‐related death problem. Missingness due to dropout and/or death is then treated as ignorable (under missingness at random and separable parameter assumptions). The estimation for the LMM is implemented by the R package nlme.

We use the Bayesian approach to obtain the partly conditional profiles of CD4 cell count given that patients were still alive. We first use the posterior samples of ***α*** ^*S*^, $\gamma_{1}^{S}$, $\gamma_{2}^{S}$ and Σ to calculate $E(b_{i1}|S_{i}⩾j,x_{ij},z_{ij})$ and $E(b_{i2}|S_{i}⩾j,x_{ij},z_{ij})$ (see more details in the on‐line supplementary materials). Then we compute $E(Y_{ij}|S_{i}⩾j,x_{ij},z_{ij})$ by using posterior samples of ***β***, $E(b_{i1}|S_{i}⩾j,x_{ij},z_{ij})$ and $E(b_{i2}|S_{i}⩾j,x_{ij},z_{ij})$. These posterior samples of $E(Y_{ij}|S_{i}⩾j,x_{ij},z_{ij})$ are used for inference on partly conditional profiles.

Theoretically, we could also sample from the asymptotic distribution of the MLEs and provide the inference of partly conditional profiles. However, because of the computational problem of obtaining the Hessian matrix in the maximum likelihood estimation for the HERS data, this approach was not pursued.

### 4.2 Model assessment

To assess the fit of the JM to the observed data, within the Bayesian estimation framework, we use posterior predictive checks based on replicated observed data as recommended in Daniels *et al*. (2012) and a *χ* ^2^ discrepancy statistic described in Gelman *et al*. (1996). Specifically, the steps are as follows.

*Step 1*: for the *i*th patient, sample a replicated dropout time $D_{i}^{\mathrm{rep}}$ from the specified dropout model, given the current posterior samples and the patient's covariate values.
*Step 2*: for the *i*th patient, sample a replicated HIV‐related death time $S_{i}^{\mathrm{rep}}$ from the specified HIV‐related death model, given the current posterior samples and the patient's covariate values.
*Step 3*: for the *i*th patient, sample the complete longitudinal outcome vector $Y_{i}^{\mathrm{rep}}$ from the specified unconditional longitudinal model, given the current posterior samples and the patient's covariate values.
*Step 4*: truncate $Y_{i}^{\mathrm{rep}}$ at the *j*th visit and $j=\min(D_{i}^{\mathrm{rep}},S_{i}^{\mathrm{rep}},12)$ to obtain the replicates of the observed longitudinal data, $Y_{i}^{o,\;\mathrm{rep}}$.
*Step 5*: repeat steps 1–4 for *N*=827 HERS patients and compute
  $$\sum_{i=1}^{827}{\left(Y_{i}^{o,\;\mathrm{rep}}-\mu_{i}\right)}^{T}\Omega^{-1}\left(Y_{i}^{o,\;\mathrm{rep}}-\mu_{i}\right)/n^{\mathrm{rep}},$$
  where *n* ^rep^ is the total number of replicates for the observed longitudinal data, ***μ***_*i*_ is the mean given in equation (10) with the random effects integrated out and Ω is the marginal covariance matrix after integrating out the random effects. Similarly, compute the *χ* ^2^‐discrepancy statistic for the observed HERS data.
*Step 6*: repeat steps 1–5 for each posterior sample and compute the posterior predictive probability that the replicated *χ* ^2^‐statistic is larger than the observed *χ* ^2^‐statistic.

The posterior probability that the *χ* ^2^‐statistic is larger than the observed *χ* ^2^‐statistic is 0.55, which indicates a reasonable fit of our JM to the observed data.

### 4.3 Results

The results for the longitudinal part of the proposed JM and the LMM are presented in Table 3. Table 2 shows the results from the dropout and survival parts of the fitted JM. We first focus on the results based on the Bayesian approach.

#### 4.3.1 Results for the unconditional longitudinal profile

The estimated main effect of time (posterior mean) in the unconditional model from the JM is −0.863 (95% credible interval [−1.105,−0.628]), which is larger in magnitude than the estimate from the LMM under ignorable missingness. The primary difference between the LMM and JM analyses is that the LMM assumes that those who dropped out or died earlier in the study had similar longitudinal CD4 cell profiles (intercept and time slopes) to those with later occurrences of these two events given past observed values and covariates. However, from Table 2 it is clear that patients who dropped out or died early tended to have larger declines in CD4 cell count over time ($\gamma_{2}^{D}=0.443$ (95% credible interval [0.349,0.537]), $\gamma_{2}^{S}=1.192$ (95% credible interval [0.911, 1.547])). As a result, the time slope under ignorable missingness may be underestimated (with a less steep decline). Similarly, the JM estimates show larger differences in the slope of CD4 cell count within baseline viral load groups, whereas Table 2 indicates that the hazards of dropout and death are both higher for those with higher baseline HIV viral load. LMM results also suggest that patients with antiretroviral therapy at baseline had a less steep decline in CD4 cell count given other covariates, but the JM analysis did not find enough evidence to support this finding. Interestingly, the estimates for the unconditional model from our JM analysis are very similar to the results from the pattern–mixture model analysis that was reported in Hogan *et al*. (2004).

**Table 3:** Results for the longitudinal part of the JM analysis and the LMM analysis (under missingness at random) of the HERS data^a^

	*Results for the JM*						*Results for the LMM*		
	*Bayesian analysis*			*MLE analysis*			*Estimate*	*2.5%*	*97.5%*
	*Mean*	*2.5%*	*97.5%*	*Estimate*	*2.5%*	*97.5%*			
Intercept	15.080	13.700	16.410	14.901	14.267	15.509	14.589	13.208	15.969
Time (visit)	−0.863	−1.105	−0.628	−0.902	−1.024	−0.795	−0.574	−0.803	−0.344
Baseline HIV viral load (copies ml^−1^)									
0–500	10.040	8.495	11.550	10.035	9.115	10.956	10.520	8.974	12.066
500–5000	6.623	5.175	8.020	6.605	5.764	7.288	6.985	5.530	8.440
5000–30000	2.977	1.464	4.519	2.946	1.907	3.907	3.210	1.611	4.808
>30000	Reference			Reference			Reference		
Baseline HIV symptoms	−0.115	−0.513	0.268	−0.182	−0.614	0.234	−0.142	−0.550	0.265
Antiretroviral therapy	−4.653	−5.485	−3.814	−4.815	−5.719	−3.990	−4.760	−5.600	−3.920
at baseline									
Time * baseline viral load (copies ml^−1^)									
0–500	0.464	0.207	0.734	0.463	0.319	0.597	0.232	−0.016	0.480
500–5000	0.433	0.183	0.684	0.422	0.311	0.533	0.220	−0.019	0.459
5000–30000	0.273	0.003	0.547	0.266	0.104	0.424	0.153	−0.108	0.414
>30000	Reference			Reference			Reference		
Time * baseline	−0.049	−0.107	0.013	−0.054	−0.117	0.010	−0.027	−0.086	0.032
HIV symptoms									
Time * antiretroviral	0.109	−0.011	0.229	0.105	−0.027	0.223	0.159	0.040	0.279
therapy at baseline									
corr(*b*_*i*1_,*b*_*i*2_)	−0.305	−0.380	−0.229	—	—	—	−0.343	—	—
var(*b*_*i*1_)	29.120	26.000	32.520	—	—	—	29.284	—	—
var(*b*_*i*2_)	0.539	0.467	0.622	—	—	—	0.450	—	—
$\sigma_{\epsilon }^{2}$	7.304	7.026	7.583	—	—	—	7.345	—	—

a For results from the Bayesian approach, we present the posterior mean, standard deviation and 95% credible intervals. For the maximum profile likelihood estimation results, we provide point estimates and 95% confidence intervals.

#### 4.3.2 Results for the conditional longitudinal profile given being alive

**Figure 2:**
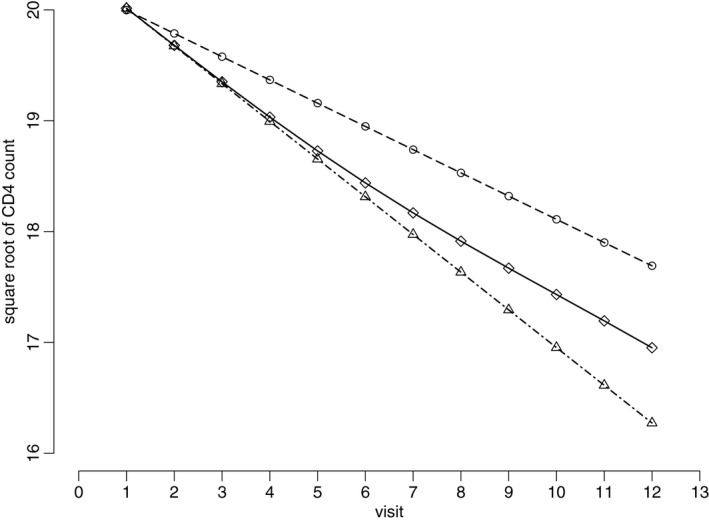
Estimated longitudinal profiles (posterior mean) of CD4 cell count for patients who had low baseline HIV viral load (0–500 copies ml^−1^), one HIV symptom and were taking antiretroviral therapy at baseline in the HERS: ∘, unconditional mean profile of CD4 cell count from the LMM analysis $\left(E\left(Y_{ij}|x_{ij}\right)=x_{ij}^{T}\beta \right)$; ▵, unconditional mean profile of CD cell count from the JM analysis $\left(E\left(Y_{ij}|x_{ij}\right)=x_{ij}^{T}\beta \right)$; ⋄, partly conditional mean profile given that the patients were still alive at the current visit $\left(E\left(Y_{ij}|x_{ij},S_{i}⩾j\right)\right)$

The inferences that are presented in Table 3 are for the unconditional model. As discussed previously, we are also interested in the conditional profiles given being alive for the longitudinal CD4 cell count outcome in the HERS. Fig. 2 presents the unconditional and partly conditional longitudinal profiles (posterior mean estimates) of CD4 cell counts for patients who had low baseline HIV viral load (0–500 copies ml^−1^), one HIV symptom and were taking antiretroviral therapy at baseline. Again, the LMM analysis underestimates the CD4 cell time slope as patients who stayed in the study tended to have a less rapid decline of CD4 cell count. The unconditional mean profile from the JM analysis corrects the selection bias but implicitly extrapolates the longitudinal CD4 cell count profile beyond death. Therefore, it gives the lowest CD4 cell count profile because it assumes that the CD4 cell count beyond death tended to be lower than those from survivors. In contrast, the partly conditional mean profile adjusts for the selection bias due to informative dropout but allows the survival differences over time. Thus it lies between the two unconditional mean profiles from the JM and LMM.

Since baseline HIV viral load is an important factor associated with informative dropout and HIV‐related deaths (Table 2), we also obtain the partly conditional mean profiles for other baseline HIV viral load groups (with one HIV symptom and antiretroviral therapy at baseline). Details can be found in the on‐line supplementary materials. As the partly conditional mean profiles take into account the survival differences over time and between baseline HIV viral load groups, the differences between the profiles from different baseline HIV viral load groups are reduced compared with those unconditional mean profiles. In other words, the interaction between time and baseline HIV viral load groups is smaller because, through the selection by survivals, the population remaining in the study is less heterogeneous.

The estimated regression coefficients from profile maximum likelihood estimation are similar to those from the Bayesian approach, which are also presented in Tables 2 and 3. However, this profile maximum likelihood approach is extremely computationally intensive as there are 32 parameters to examine. To make the computational time affordable, we stop the program at 500 iterations to obtain the maximum profile likelihood estimates at each grid point of the parameters. In Tables 2 and 3, we find that most confidence intervals from the maximum profile likelihood approach are narrower than the credible intervals that are obtained from the Bayesian approach, which is possibly due to the cut‐off of 500 iterations as suggested by our investigations.

#### 4.3.3 Summarizing remarks

Overall, our results for the unconditional model are consistent with the findings that were reported in Hogan *et al*. (2004), i.e. baseline HIV viral load groups had very different unconditional CD4 cell count profiles over time. However, these differences were smaller if we focus on the conditional CD4 cell count profiles of the survivor population over time, which was not provided in the pattern–mixture model approach in Hogan *et al*. (2004). In addition, we find that baseline HIV viral load groups were associated with dropout and HIV‐related death, and baseline antiretroviral therapy status was associated with HIV‐related death, whereas these factors that were related to the selection processes of dropout and HIV‐related death were not able to be examined in Hogan *et al*. (2004).

## 5 Simulation study

In this section, we conduct a brief simulation study to examine the finite sample performance of the JM proposed. The design of the simulation study is motivated by the HERS analysis in Section 4. We perform the estimation by using the Bayesian approach, because of the computational issues from the maximum likelihood approach as discussed in Section 4. Details about the simulation design and estimation can be found in the on‐line supplementary materials.

For regression coefficients and variance component parameters in the longitudinal model (Table 1 in the on‐line supplementary materials), the posterior mean estimates based on the JM proposed had minimal absolute biases and the 95% credible intervals had good coverage probabilities.

Overall, the posterior mean estimates from the dropout model had small biases and the 95% credible intervals had good coverage probabilities (Table 2 in the on‐line supplementary materials). For the HIV‐related death model, the biases are slightly larger for certain parameters, possibly because of the large curvature in the true regression function as well as the high survival probabilities in this simulation design and the consequent lack of information from the data for estimation. In addition, compared with the parameters in the dropout model, the parameter estimates in the HIV‐related death model had larger empirical standard deviations. Further discussions about the simulation results can be found in the supplementary materials.

## 6 Discussion

In this paper, we developed a new likelihood‐based approach to dealing with informative dropout mixed with death in longitudinal studies. An important feature of our approach is that inferences from both unconditional and partly conditional models can be obtained conveniently. Moreover, compared with the existing weighted estimating equation approaches for partly conditional models, our likelihood‐based approach explicitly allows for informative dropout, censoring of survival information and also intermittent missing data. Our model also helps to shed light on the factors that influence the selection process by dropout and survival. The HERS data analysis in Section 4 confirmed the findings in Hogan *et al*. (2004) for the unconditional model, but it also provided the inferences for partly conditional models, which were not addressed in the pattern–mixture model approach of Hogan *et al*. (2004).

In our motivating application from the HERS, we focus on the longitudinal outcome; and HIV disease progression represented by changes in CD4 cell count is believed to be strongly associated with the dropout and HIV‐related death. Therefore, we use random effects in the model for CD4 cell counts to characterize the HIV disease progression, and govern the relationships between HIV disease progression and dropout and HIV‐related death. A key assumption in our joint modelling approach is the independence of the *complete* longitudinal data and semicompeting risks data given the random effects and covariates. As suggested by a referee, to assess the conditional independence between dropout and death times we could introduce additional frailty terms apart from the random effects that are specified in the longitudinal model. However, one aspect of the conditional independence assumption is unverifiable because we cannot assess the conditional independence between *missing* longitudinal data and dropout and death times given random effects and covariates. In practice, we could carefully model the residual covariance and random‐effects structures in the JM such that the valid extrapolation of the missing longitudinal data is more plausible. As in all problems with informative missing data, a sensitivity analysis is required to check the effect of the unverifiable assumption about the extrapolation of the missing data on the inferences and conclusions drawn from the models fitted to the observed data. Unfortunately, unlike selection models and pattern–mixture models for addressing informative missing data problems, research for sensitivity analysis strategies under the shared parameter model framework is very limited and it is not clear how to perform sensitivity analyses without changing the inferences based on the observed data (Daniels and Hogan (2008), chapter 8). Further work on sensitivity analysis within the shared parameter model framework is of great interest.

Following the literature for the joint modelling of longitudinal and time‐to‐event data, we specified a parametric LMM for the unconditional model of the longitudinal outcome. This can be made more flexible by incorporating splines or fractional polynomials in both the population level and the individual level longitudinal profiles. Therefore, the serial correlation can be more flexibly characterized with a time varying random‐effects specification; and the functional forms of the regression function in the longitudinal part can be made semiparametric. In addition, more flexible functional forms can be introduced into the regression models for dropout and HIV‐related death to relax the parametric assumption.

We emphasize, regardless of how flexibly we specify the joint model, extrapolation is always involved when making inferences about the unconditional longitudinal outcome profile by using data truncated by dropout and death. We could assess only whether the model fits the observed data well (Daniels and Hogan, 2008), which is an advantage of our methods because model assessment based on the observed data is more straightforward within the likelihood‐based frameworks (e.g. through the posterior predictive checks in the Bayesian framework).

As pointed out by a referee, a longitudinal model with ignorable dropout and death times is also an option in practice. When all covariates that are associated with the dropout and death times are included in the longitudinal model and the random effects that characterize the disease progression are not associated with the dropout and death times, the longitudinal outcome is independent of the dropout and death times given covariates. In this case, the dropout and death times are ignorable, given that they have distinct parameters from the parameters of the longitudinal model and the longitudinal model (including the covariance structure) is correctly specified (Little and Rubin, 2002; Daniels and Hogan, 2008). Under this model, the mean of the longitudinal outcome conditionally on being alive is the same as the unconditional mean, given covariates. Our approach relaxes the assumption in this model with ignorable dropout and death times by allowing the random effects that characterize the underlying disease progression to be associated with the dropout and death times, although we still make unverifiable assumptions as discussed. In practice, it is difficult to differentiate the models assuming ignorable missingness and non‐ignorable missingness on the basis of the model fits to the observed data only (Molenberghs *et al*., 2008). Therefore, it is important to include the model with ignorable dropout and death times as a plausible option. Note that the key requirements under this model are inclusion of all covariates that are associated with dropout and death and correct specification of the full distribution for the longitudinal outcome.

In the HERS data, the observed dropout time is discrete. We discretize the time for HIV‐related death following Barrett *et al*. (2015) such that the dropout and HIV‐related death follow the same timescale. Barrett *et al*. (2015) investigated the effect of discretization of the timescale on the inferences of the longitudinal and survival submodels. Their simulation studies and analysis of special cases suggested that the parameter estimates (in particular, the covariate effects in the longitudinal and survival submodels) were not greatly influenced by the discretization. Moreover, Barrett *et al*. (2015) theoretically proved that there is no loss of information when the survival functions are linear between discrete time points. In practice, a discretization that ensures approximate linearity was recommended.

The Bayesian approach for fitting our model is relatively straightforward and the WinBUGS code and R code for obtaining partly conditional profiles are available from http://wileyonlinelibrary.com/journal/rss-datasets.

Owing to the complexity of derivatives of multivariate normal distribution functions with respect to the unknown parameters, the maximum likelihood estimation and Hessian matrix can be obtained only numerically, and we had computational issues in implementing the maximum likelihood estimation for the HERS analysis. As mentioned previously, the challenges include the following.

- The estimation and inference are extremely computationally intensive because of the need to calculate the multivariate normal probabilities of high dimension, together with the large number of model parameters.
- Monte Carlo errors are unavoidable when approximating multivariate normal probabilities of high dimensions in the evaluations of the log‐likelihood function.

These errors not only introduce noise in the convergence process when it reaches the neighbourhood of the MLE but also lead to inaccurate numerical approximation of the Hessian matrix. In our investigation for the analysis of HERS data, the R function optim with the Nelder–Mead method struggles to find the parameter values that minimize the minus log‐likelihood function after 5000 iterations. It appears that it is impossible to reach a reasonably small tolerance for convergence. These Monte Carlo errors are more prominent in our analysis than the analysis in Barrett *et al*. (2015) probably because of the calculation of higher dimensional (up to 24) multivariate normal probabilities in our model. A possible solution to this problem is the maximum smoothed likelihood estimation (Ionides, 2005). In fact, our profile maximum likelihood approach for the HERS data has partly used this concept of smoothing the approximated log‐likelihood functions. Further research is required on the use of maximum smoothed likelihood estimation for our JM. Directly calculating the conditional mean of the random effects given being alive in Section 3.4 also involves calculation of multivariate normal probabilities. In the HERS application, the situation is slightly better than calculating the marginal likelihood, because the dimension is up to 12 (instead of 24). Another approach to obtaining the conditional mean of random effects given being alive is to sample directly from the multivariate skew normal distribution for the random effects, given the posterior samples of the model parameters, and to calculate the corresponding sample means of the random‐effects samples. This sampling procedure is made easier because of the specification of our JM under discretization of the death timescale. For other JMs with continuous timescale for death, the conditional distribution of random effects given being alive will usually not have a closed form. Therefore, the sampling will require more computational steps, for example, through the Metropolis–Hastings algorithm (Rizopoulos, 2011). As pointed out by a referee, when the dimension of the random effect is low (e.g. with random intercept and slope only), Gauss–Hermite quadrature can be used to integrate out random effects for calculating the marginal likelihood directly. In this case, the above computational issue for calculating multivariate normal probabilities does not apply to fitting our JM. When the dimension of random effects is high (e.g. with time varying random effects through specification of splines), it is challenging to approximate effectively the integration by using Gaussian quadrature. Overall, in practice we recommend using the Bayesian approach for estimation in our JM, because of its computational efficiency with off‐the‐shelf software and the challenges in maximum likelihood estimation when calculating high dimensions of multivariate normal probabilities is required.

## Supporting information

‘Supplementary Materials for “Accommodating informative dropout and death: a joint modelling approach for longitudinal and semi‐competing risks data”’.
