# Supplementary material for: Accommodating informative dropout and death: a joint modelling approach for longitudinal and semicompeting risks data
Source: J R Stat Soc Ser C Appl Stat. 2017 Jan 30;67(1):145–63. doi: 10.1111/rssc.12210 (PMC5741179; doi:10.1111/rssc.12210)
Supplement: Supplementary file 1 — ‘Supplementary Materials for “Accommodating informative dropout and death: a joint modelling approach for longitudinal and semi‐competing risks data”’. [file RSSC-67-145-s001.pdf]

Supplementary Materials for 'Accommodating informative dropout and death: a joint modelling approach for longitudinal and semi-competing risks data'

Qiuju Li and Li Su*

Medical Research Council Biostatistics Unit, University of Cambridge

1 Marginal likelihood

Using the multivariate skew-normal distribution results (Arnold, 2009; Barrett *et al.*, 2015), it can be shown that the random effects in the likelihood in (4) of the main text can be integrated out and the marginal likelihood can be written in a closed form using well-defined functions.

Let $H_i = Z_i^{\mathrm{T}} V_i^{-1} Z_i + \Sigma^{-1}$, $\mathbf{h}_i = H_i^{-1} Z_i^{\mathrm{T}} V_i^{-1}(\mathbf{Y}_i^o - X_i^{\mathrm{T}}\boldsymbol{\beta})$ and

$$\begin{aligned}\mathcal{L}_{1i}(\boldsymbol{\theta} \mid \mathbf{Y}_i^o) &= \exp\Big\{ -\log(2\pi)n_i/2 - \log(|V_i|)/2 - \log(|\Sigma H_i|)/2 \\ &\quad -(\mathbf{Y}_i^o - X_i\boldsymbol{\beta})^{\mathrm{T}} V_i^{-1}(\mathbf{Y}_i^o - X_i\boldsymbol{\beta})/2 + \mathbf{h}_i^{\mathrm{T}} H_i \mathbf{h}_i/2\Big\}. \end{aligned} \quad (1)$$

Then we have

$$f(\mathbf{Y}_i^o \mid \mathbf{b}_i; \boldsymbol{\theta}) f(\mathbf{b}_i; \boldsymbol{\theta}) = \phi^{(q)}(\mathbf{b}_i; \mathbf{h}_i, H_i^{-1}) \mathcal{L}_{1i}(\boldsymbol{\theta} \mid \mathbf{Y}_i^o),$$

where $\phi^{(q)}(\mathbf{b}_i; \mathbf{h}_i, H_i^{-1})$ is the density function of q-dimensional multivariate normal distribution with mean $\mathbf{h}_i$ and covariance matrix H_i^{-1}. Further, we define $X_{D,ij} = (\mathbf{x}_{D,i1}, \dots, \mathbf{x}_{D,ij})^{\mathrm{T}}$, $L_{ij}^D = (W_{D,i1}^{\mathrm{T}}\boldsymbol{\gamma}_1^D, \dots, W_{D,ij}^{\mathrm{T}}\boldsymbol{\gamma}_j^D)^{\mathrm{T}}$ for $j = 1, \dots, d$, $X_{S,ij} = (\mathbf{x}_{S,i1}, \dots, \mathbf{x}_{S,ij})^{\mathrm{T}}$ and $L_{ij}^S =$

*li.su@mrc-bsu.cam.ac.uk; MRC Biostatistics Unit, University of Cambridge, Robinson Way, Cambridge CB2 0SR, UK

$(W_{S,i1}^{\mathrm{T}}\boldsymbol{\gamma}_1^S, \ldots, W_{S,ij}^{\mathrm{T}}\boldsymbol{\gamma}_j^S)^{\mathrm{T}}$ for $j = 1, \ldots, s$. Let $\Phi^{(j)}(\cdot; \boldsymbol{\mu}, \Delta)$ be the j-dimensional multivariate normal cumulative distribution function with mean $\boldsymbol{\mu}$ and covariance matrix Δ, and we denote $\Phi^{(j)}(\cdot) = \Phi^{(j)}(\cdot; \mathbf{0}, I)$.

There are 4 scenarios for $f(d, \delta_i^D, s, \delta_i^S \mid \mathbf{b}_i; \boldsymbol{\theta})$:

(1) When $\delta_i^D = 0$ and $\delta_i^S = 0$ such that $d = s = C_i$,

$$\begin{aligned} & f(d, \delta_i^D, s, \delta_i^S \mid \mathbf{b}_i; \boldsymbol{\theta}) \\ = \; & \Phi^{(d)}(X_{D,id}\boldsymbol{\alpha}^D + L_{id}^D\mathbf{b}_i)\Phi^{(s)}(X_{S,is}\boldsymbol{\alpha}^S + L_{is}^S\mathbf{b}_i). \end{aligned}$$

(2) When $\delta_i^D = 1$ and $\delta_i^S = 0$ such that $d \le s = C_i$,

$$\begin{aligned} & f(d, \delta_i^D, s, \delta_i^S \mid \mathbf{b}_i; \boldsymbol{\theta}) \\ = \; & \left\{\Phi^{(d-1)}(X_{D,i(d-1)}\boldsymbol{\alpha}^D + L_{i(d-1)}^D\mathbf{b}_i) - \Phi^{(d)}(X_{D,id}\boldsymbol{\alpha}^D + L_{id}^D\mathbf{b}_i)\right\}\Phi^{(s)}(X_{S,is}\boldsymbol{\alpha}^S + L_{is}^S\mathbf{b}_i). \end{aligned}$$

(3) When $\delta_i^D = 0$ and $\delta_i^S = 1$ such that $d = s \le C_i$,

$$\begin{aligned} & f(d, \delta_i^D, s, \delta_i^S \mid \mathbf{b}_i; \boldsymbol{\theta}) \\ = \; & \Phi^{(d)}(X_{D,id}\boldsymbol{\alpha}^D + L_{id}^D\mathbf{b}_i)\left\{\Phi^{(s-1)}(X_{S,i(s-1)}\boldsymbol{\alpha}^S + L_{i(s-1)}^S\mathbf{b}_i) - \Phi^{(s)}(X_{S,is}\boldsymbol{\alpha}^S + L_{is}^S\mathbf{b}_i)\right\}. \end{aligned}$$

(4) When $\delta_i^D = 1$ and $\delta_i^S = 1$ such that $d \le s \le C_i$,

$$\begin{aligned} & f(d, \delta_i^D, s, \delta_i^S \mid \mathbf{b}_i; \boldsymbol{\theta}) \\ = \; & \left\{\Phi^{(d-1)}(X_{D,i(d-1)}\boldsymbol{\alpha}^D + L_{i(d-1)}^D\mathbf{b}_i) - \Phi^{(d)}(X_{D,id}\boldsymbol{\alpha}^D + L_{id}^D\mathbf{b}_i)\right\} \\ \times \; & \left\{\Phi^{(s-1)}(X_{S,i(s-1)}\boldsymbol{\alpha}^S + L_{i(s-1)}^S\mathbf{b}_i) - \Phi^{(s)}(X_{S,is}\boldsymbol{\alpha}^S + L_{is}^S\mathbf{b}_i)\right\}. \end{aligned}$$

Next we write

$$\begin{aligned} \mathcal{L}_i(\boldsymbol{\theta} \mid \mathbf{Y}_i^o, D_i^* = d, \delta_i^D, S_i^* = s, \delta_i^S) &= \int \mathcal{L}_i(\boldsymbol{\theta} \mid \mathbf{Y}_i^o, D_i^* = d, \delta_i^D, S_i^* = s, \delta_i^S, \mathbf{b}_i)d\mathbf{b}_i \\ &= \mathcal{L}_{1i}(\boldsymbol{\theta} \mid \mathbf{Y}_i^o)\mathcal{L}_{2i}(\boldsymbol{\theta} \mid \mathbf{Y}_i^o, d, \delta_i^D, s, \delta_i^S), \end{aligned}$$

where

$$\mathcal{L}_{2i}(\boldsymbol{\theta} \mid \mathbf{Y}_i^o, d, \delta_i^D, s, \delta_i^S) = \int f(d, \delta_i^D, s, \delta_i^S \mid \mathbf{b}_i; \boldsymbol{\theta})\phi^{(q)}(\mathbf{b}_i; \mathbf{h}_i, H_i^{-1})d\mathbf{b}_i.$$

Let

$$\mathbf{A}_{i,ds} = \begin{bmatrix} X_{D,id}\boldsymbol{\alpha}^D \\ X_{S,is}\boldsymbol{\alpha}^S \end{bmatrix}, \mathbf{A}_{i,(d-1)s} = \begin{bmatrix} X_{D,i(d-1)}\boldsymbol{\alpha}^D \\ X_{S,is}\boldsymbol{\alpha}^S \end{bmatrix},$$
$$\mathbf{A}_{i,d(s-1)} = \begin{bmatrix} X_{D,id}\boldsymbol{\alpha}^D \\ X_{S,i(s-1)}\boldsymbol{\alpha}^S \end{bmatrix}, \mathbf{A}_{i,(d-1)(s-1)} = \begin{bmatrix} X_{D,i(d-1)}\boldsymbol{\alpha}^D \\ X_{S,i(s-1)}\boldsymbol{\alpha}^S \end{bmatrix},$$
$$\mathbf{B}_{i,ds} = \begin{bmatrix} L_{id}^D \\ L_{is}^S \end{bmatrix}, \mathbf{B}_{i,(d-1)s} = \begin{bmatrix} L_{i(d-1)}^D \\ L_{is}^S \end{bmatrix},$$
$$\mathbf{B}_{i,d(s-1)} = \begin{bmatrix} L_{id}^D \\ L_{i(s-1)}^S \end{bmatrix}, \mathbf{B}_{i,(d-1)(s-1)} = \begin{bmatrix} L_{i(d-1)}^D \\ L_{i(s-1)}^S \end{bmatrix}.$$

Using the multivariate skew-normal distribution results (Arnold, 2009; Barrett *et al.*, 2015) and the property of $\int_{-\infty}^{\infty} f(\mathbf{x})d\mathbf{x} = 1$ if $f(\mathbf{x})$ is a probability density function, it can be shown that

(1) when $\delta_i^D = 0$ and $\delta_i^S = 0$ such that $d = s = C_i$,

$$\begin{aligned} &\mathcal{L}_{2i}(\boldsymbol{\theta} \mid \mathbf{Y}_i^o, d, \delta_i^D, s, \delta_i^S) \\ = \; & \Phi^{(d+s)}\left(\mathbf{A}_{i,ds} + \mathbf{B}_{i,ds}\mathbf{h}_i; \mathbf{0}, I + \mathbf{B}_{i,ds}H_i^{-1}\mathbf{B}_{i,ds}^{\mathrm{T}}\right); \end{aligned}$$

(2) when $\delta_i^D = 1$ and $\delta_i^S = 0$ such that $d \le s \le C_i$,

$$\begin{aligned} &\mathcal{L}_{2i}(\boldsymbol{\theta} \mid \mathbf{Y}_i^o, d, \delta_i^D, s, \delta_i^S) \\ = \; & \Phi^{(d-1+s)}\left(\mathbf{A}_{i,(d-1)s} + \mathbf{B}_{i,(d-1)s}\mathbf{h}_i; \mathbf{0}, I + \mathbf{B}_{i,(d-1)s}H_i^{-1}\mathbf{B}_{i,(d-1)s}^{\mathrm{T}}\right) \\ & -\Phi^{(d+s)}\left(\mathbf{A}_{i,ds} + \mathbf{B}_{i,ds}\mathbf{h}_i; \mathbf{0}, I + \mathbf{B}_{i,ds}H_i^{-1}\mathbf{B}_{i,ds}^{\mathrm{T}}\right); \end{aligned}$$

(3) when $\delta_i^D = 0$ and $\delta_i^S = 1$ such that $d = s \le C_i$,

$$\begin{aligned} &\mathcal{L}_{2i}(\boldsymbol{\theta} \mid \mathbf{Y}_i^o, d, \delta_i^D, s, \delta_i^S) \\ = \; & \Phi^{(d+s-1)}\left(\mathbf{A}_{i,d(s-1)} + \mathbf{B}_{i,d(s-1)}\mathbf{h}_i; \mathbf{0}, I + \mathbf{B}_{i,d(s-1)}H_i^{-1}\mathbf{B}_{i,d(s-1)}^{\mathrm{T}}\right) \\ & -\Phi^{(d+s)}\left(\mathbf{A}_{i,ds} + \mathbf{B}_{i,ds}\mathbf{h}_i; \mathbf{0}, I + \mathbf{B}_{i,ds}H_i^{-1}\mathbf{B}_{i,ds}^{\mathrm{T}}\right); \end{aligned}$$

(4) when $\delta_i^D = 1$ and $\delta_i^S = 1$ such that $d \le s \le C_i$,

$$
\begin{aligned}
&\mathcal{L}_{2i}(\boldsymbol{\theta} \mid \mathbf{Y}_i^o, d, \delta_i^D, s, \delta_i^S) \\
= \; & \Phi^{(d-1+s-1)}\left(\mathbf{A}_{i,(d-1)(s-1)} + \mathbf{B}_{i,(d-1)(s-1)}\mathbf{h}_i; \mathbf{0}, I + \mathbf{B}_{i,(d-1)(s-1)} H_i^{-1} \mathbf{B}_{i,(d-1)(s-1)}^{\mathrm{T}}\right) \\
& -\Phi^{(d-1+s)}\left(\mathbf{A}_{i,(d-1)s} + \mathbf{B}_{i,(d-1)s}\mathbf{h}_i; \mathbf{0}, I + \mathbf{B}_{i,(d-1)s} H_i^{-1} \mathbf{B}_{i,(d-1)s}^{\mathrm{T}}\right) \\
& -\Phi^{(d+s-1)}\left(\mathbf{A}_{i,d(s-1)} + \mathbf{B}_{i,d(s-1)}\mathbf{h}_i; \mathbf{0}, I + \mathbf{B}_{i,d(s-1)} H_i^{-1} \mathbf{B}_{i,d(s-1)}^{\mathrm{T}}\right) \\
& +\Phi^{(d+s)}\left(\mathbf{A}_{i,ds} + \mathbf{B}_{i,ds}\mathbf{h}_i; \mathbf{0}, I + \mathbf{B}_{i,ds} H_i^{-1} \mathbf{B}_{i,ds}^{\mathrm{T}}\right).
\end{aligned}
$$

When the dimension j of $\Phi^{(j)}(\cdot; \boldsymbol{\mu}, \mathit{\Delta})$ does not exceed 20, we use the `R` package `mnormt` for numerical evaluation of the multivariate normal probabilities in $\mathcal{L}_{2i}(\boldsymbol{\theta} \mid \mathbf{Y}_i, s, \delta_i)$ in the HERS data analysis. If $j > 20$, we use the `R` package `mvtnorm`.

2 Mean of the conditional distribution of $\mathbf{b}_i$ given $S_i \ge j$

The conditional density of $\mathbf{b}_i$ given $S_i \ge j$ (and $X_{S,i(j-1)}$ implicitly) is

$$
f(\mathbf{b}_i \mid S_i \ge j) \propto \phi^{(q)}(\mathbf{b}_i; \mathbf{0}, \mathit{\Sigma})\Phi^{(j-1)}\left(X_{S,i(j-1)}\boldsymbol{\alpha}^S + L_{i(j-1)}^S \mathbf{b}_i\right),
$$

which follows the form of a multivariate skew-normal distribution (Arnold, 2009). Specifically,

$$
\begin{aligned}
& f(\mathbf{b}_i \mid S_i \ge j) \qquad (2)\\
= \; & \phi^{(q)}(\mathbf{b}_i; \mathbf{0}, \mathit{\Sigma}) \frac{\Phi^{(j-1)}\left(X_{S,i(j-1)}\boldsymbol{\alpha}^S + L_{i(j-1)}^S \mathbf{b}_i; \mathbf{0}, I\right)}{\Phi^{(j-1)}\left(X_{S,i(j-1)}\boldsymbol{\alpha}^S; \mathbf{0}, I + L_{i(j-1)}^S \mathit{\Sigma} (L_{i(j-1)}^S)^{\mathrm{T}}\right)}
\end{aligned}
$$

To calculate the mean of multivariate skew-normal distribution, we first derive its moment generating function (González-Farías *et al.*, 2004; Flecher *et al.*, 2009) as follows,

$$M(\mathbf{t}) = \mathrm{E}(e^{\mathbf{t}^{\mathrm{T}}\mathbf{b}_i}|S_i \geq j) = \int_{-\infty}^{\infty} e^{\mathbf{t}^{\mathrm{T}}\mathbf{b}_i} f(\mathbf{b}_i|S_i \geq j)d\mathbf{b}_i$$

$$= \int_{-\infty}^{\infty} e^{\mathbf{t}^{\mathrm{T}}\mathbf{b}_i} \phi^{(q)}(\mathbf{b}_i; \mathbf{0}, \Sigma) \frac{\Phi^{(j-1)}\left(X_{S,i(j-1)}\boldsymbol{\alpha}^S + L^S_{i(j-1)}\mathbf{b}_i; \mathbf{0}, I\right)}{\Phi^{(j-1)}\left(X_{S,i(j-1)}\boldsymbol{\alpha}^S; \mathbf{0}, I + L^S_{i(j-1)}\Sigma(L^S_{i(j-1)})^{\mathrm{T}}\right)} d\mathbf{b}_i$$

$$= \int_{-\infty}^{\infty} e^{\mathbf{t}^{\mathrm{T}}\mathbf{b}_i} \exp\left\{ -\log(2\pi)q/2 - \log(|\Sigma|)/2 - \mathbf{b}_i^{\mathrm{T}}\Sigma^{-1}\mathbf{b}_i/2 \right\}$$

$$\times \frac{\Phi^{(j-1)}\left(X_{S,i(j-1)}\boldsymbol{\alpha}^S + L^S_{i(j-1)}\mathbf{b}_i; \mathbf{0}, I\right)}{\Phi^{(j-1)}\left(X_{S,i(j-1)}\boldsymbol{\alpha}^S; \mathbf{0}, I + L^S_{i(j-1)}\Sigma(L^S_{i(j-1)})^{\mathrm{T}}\right)} d\mathbf{b}_i$$

$$= e^{\frac{1}{2}\mathbf{t}^{\mathrm{T}}\Sigma\mathbf{t}} \int_{-\infty}^{\infty} \exp\left\{ -\log(2\pi)q/2 - \log(|\Sigma|)/2 - (\mathbf{b}_i - \Sigma\mathbf{t})^{\mathrm{T}}\Sigma^{-1}(\mathbf{b}_i - \Sigma\mathbf{t})/2 \right\}$$

$$\times \frac{\Phi^{(j-1)}\left(X_{S,i(j-1)}\boldsymbol{\alpha}^S + L^S_{i(j-1)}\mathbf{b}_i; \mathbf{0}, I\right)}{\Phi^{(j-1)}\left(X_{S,i(j-1)}\boldsymbol{\alpha}^S; \mathbf{0}, I + L^S_{i(j-1)}\Sigma(L^S_{i(j-1)})^{\mathrm{T}}\right)} d\mathbf{b}_i$$

$$= e^{\frac{1}{2}\mathbf{t}^{\mathrm{T}}\Sigma\mathbf{t}} \int_{-\infty}^{\infty} \phi^{(q)}(\mathbf{b}_i; \Sigma\mathbf{t}, \Sigma) \frac{\Phi^{(j-1)}\left(X_{S,i(j-1)}\boldsymbol{\alpha}^S + L^S_{i(j-1)}\Sigma\mathbf{t} + L^S_{i(j-1)}(\mathbf{b}_i - \Sigma\mathbf{t}); \mathbf{0}, I\right)}{\Phi^{(j-1)}\left(X_{S,i(j-1)}\boldsymbol{\alpha}^S + L^S_{i(j-1)}\Sigma\mathbf{t}; \mathbf{0}, I + L^S_{i(j-1)}\Sigma(L^S_{i(j-1)})^{\mathrm{T}}\right)} d\mathbf{b}_i$$

$$\times \frac{\Phi^{(j-1)}\left(X_{S,i(j-1)}\boldsymbol{\alpha}^S + L^S_{i(j-1)}\Sigma\mathbf{t}; \mathbf{0}, I + L^S_{i(j-1)}\Sigma(L^S_{i(j-1)})^{\mathrm{T}}\right)}{\Phi^{(j-1)}\left(X_{S,i(j-1)}\boldsymbol{\alpha}^S; \mathbf{0}, I + L^S_{i(j-1)}\Sigma(L^S_{i(j-1)})^{\mathrm{T}}\right)}$$

$$= e^{\frac{1}{2}\mathbf{t}^{\mathrm{T}}\Sigma\mathbf{t}} \frac{\Phi^{(j-1)}\left(L^S_{i(j-1)}\Sigma\mathbf{t}; -X_{S,i(j-1)}\boldsymbol{\alpha}^S, I + L^S_{i(j-1)}\Sigma(L^S_{i(j-1)})^{\mathrm{T}}\right)}{\Phi^{(j-1)}\left(\mathbf{0}; -X_{S,i(j-1)}\boldsymbol{\alpha}^S, I + L^S_{i(j-1)}\Sigma(L^S_{i(j-1)})^{\mathrm{T}}\right)},$$

since

$$\int_{-\infty}^{\infty} \phi^{(q)}(\mathbf{b}_i; \Sigma\mathbf{t}, \Sigma) \frac{\Phi^{(j-1)}\left(X_{S,i(j-1)}\boldsymbol{\alpha}^S + L^S_{i(j-1)}\Sigma\mathbf{t} + L^S_{i(j-1)}(\mathbf{b}_i - \Sigma\mathbf{t}); \mathbf{0}, I\right)}{\Phi^{(j-1)}\left(X_{S,i(j-1)}\boldsymbol{\alpha}^S + L^S_{i(j-1)}\Sigma\mathbf{t}; \mathbf{0}, I + L^S_{i(j-1)}\Sigma(L^S_{i(j-1)})^{\mathrm{T}}\right)} d\mathbf{b}_i = 1$$

due to the multivariate skew-normal results.

Then we have,

$$
\begin{aligned}
\mathrm{E}(\mathbf{b}_i|S_i \geq j) &= \frac{\partial M(\mathbf{t})}{\partial \mathbf{t}}|_{\mathbf{t}=\mathbf{0}} \\
&= \frac{\Phi^{(j-1)}\left(L^S_{i(j-1)}\Sigma\mathbf{t}; -X_{S,i(j-1)}\boldsymbol{\alpha}^S, I + L^S_{i(j-1)}\Sigma(L^S_{i(j-1)})^{\mathrm{T}}\right)}{\Phi^{(j-1)}\left(\mathbf{0}; -X_{S,i(j-1)}\boldsymbol{\alpha}^S, I + L^S_{i(j-1)}\Sigma(L^S_{i(j-1)})^{\mathrm{T}}\right)} e^{\frac{1}{2}\mathbf{t}^{\mathrm{T}}\Sigma\mathbf{t}}\Sigma\mathbf{t}\ |_{\mathbf{t}=\mathbf{0}} \\
&\quad + \frac{\Phi^{(j-1)*}\left(L^S_{i(j-1)}\Sigma\mathbf{t}; -X_{S,i(j-1)}\boldsymbol{\alpha}^S, I + L^S_{i(j-1)}\Sigma(L^S_{i(j-1)})^{\mathrm{T}}\right)}{\Phi^{(j-1)}\left(\mathbf{0}; -X_{S,i(j-1)}\boldsymbol{\alpha}^S, I + L^S_{i(j-1)}\Sigma(L^S_{i(j-1)})^{\mathrm{T}}\right)} e^{\frac{1}{2}\mathbf{t}^{\mathrm{T}}\Sigma\mathbf{t}}\ |_{\mathbf{t}=\mathbf{0}} \\
&= \frac{\Phi^{(j-1)*}\left(L^S_{i(j-1)}\Sigma\mathbf{t}; -X_{S,i(j-1)}\boldsymbol{\alpha}^S, I + L^S_{i(j-1)}\Sigma(L^S_{i(j-1)})^{\mathrm{T}}\right)}{\Phi^{(j-1)}\left(\mathbf{0}; -X_{S,i(j-1)}\boldsymbol{\alpha}^S, I + L^S_{i(j-1)}\Sigma(L^S_{i(j-1)})^{\mathrm{T}}\right)}\ |_{\mathbf{t}=\mathbf{0}},
\end{aligned}
$$

where

$$
\begin{aligned}
&\Phi^{(j-1)*}\left(L^S_{i(j-1)}\Sigma\mathbf{t}; -X_{S,i(j-1)}\boldsymbol{\alpha}^S, I + L^S_{i(j-1)}\Sigma(L^S_{i(j-1)})^{\mathrm{T}}\right) \\
=&\frac{\partial}{\partial \mathbf{t}}\Phi^{(j-1)}\left(L^S_{i(j-1)}\Sigma\mathbf{t}; -X_{S,i(j-1)}\boldsymbol{\alpha}^S, I + L^S_{i(j-1)}\Sigma(L^S_{i(j-1)})^{\mathrm{T}}\right) \\
=&\frac{\partial}{\partial \mathbf{t}}\int_{-\infty}^{L^S_{i(j-1)}\Sigma\mathbf{t}} f(z_1, \cdots, z_{j-1})dz_1 \cdots dz_{j-1} = \frac{\partial}{\partial \mathbf{t}}\int_{-\infty}^{L^S_{i(j-1)}\Sigma\mathbf{t}} f(\mathbf{z})d\mathbf{z}
\end{aligned} \tag{3}
$$

and the vector $\mathbf{z} = (z_1, \ldots, z_{j-1})^{\mathrm{T}}$ follows a multivariate normal distribution with mean $\tilde{\boldsymbol{\mu}}_{ij} = -X_{S,i(j-1)}\boldsymbol{\alpha}^S$ and covariance matrix $\tilde{\Sigma}_{ij} = I + L^S_{i(j-1)}\Sigma(L^S_{i(j-1)})^{\mathrm{T}}$.

We further denote $[\mathbf{u}]_k$ the kth element of vector $\mathbf{u}$, $[\mathbf{u}]_{-k}$ the vector of $\mathbf{u}$ after removing the kth element, $[A]_{k,\cdot}$ the kth row vector of matrix A, $[A]_{-k,\cdot}$ the matrix A after removing the kth row vector, $[A]_{k,\ell}$ the (k, ℓ)th element of matrix A, $[A]_{-k,\ell}$ the ℓth column vector of matrix A after further deleting the kth element, $[A]_{k,-\ell}$ the kth row vector of matrix A after further deleting the ℓth element, $[A]_{-k,-\ell}$ the matrix A after deleting the kth row and the ℓth column.

Since $\mathbf{z} \sim N(\tilde{\boldsymbol{\mu}}_{ij}, \tilde{\Sigma}_{ij})$, we have the conditional distribution of $[\mathbf{z}]_{-k}$ given z_k, $[\mathbf{z}]_{-k} \mid z_k \sim N\Big(\tilde{\boldsymbol{\mu}}_{ij|k}(z_k), \tilde{\Sigma}_{ij|k}\Big)$ $(k = 1, \ldots, j-1)$ with

$$
\begin{aligned}
\tilde{\boldsymbol{\mu}}_{ij|k}(z_k) &= [\tilde{\boldsymbol{\mu}}_{ij}]_{-k} + [\tilde{\Sigma}_{ij}]_{-k,k}([\tilde{\Sigma}_{ij}]_{k,k})^{-1}(z_k - [\tilde{\boldsymbol{\mu}}_{ij}]_k), \\
\tilde{\Sigma}_{ij|k} &= [\tilde{\Sigma}_{ij}]_{-k,-k} - [\tilde{\Sigma}_{ij}]_{-k,k}([\tilde{\Sigma}_{ij}]_{k,k})^{-1}[\tilde{\Sigma}_{ij}]_{k,-k}.
\end{aligned}
$$

Thus the ℓth element of differentiation in (3) is,

$$\frac{\partial}{\partial \mathbf{t}_\ell}\int_{-\infty}^{L^S_{i(j-1)}\Sigma\mathbf{t}} f(z_1,\cdots,z_{j-1})dz_1\cdots dz_{j-1}$$

$$=\sum_{k=1}^{j-1}\frac{\partial}{\partial\left(\left[L^S_{i(j-1)}\Sigma\right]_{k,.}\mathbf{t}\right)}\left(\int_{-\infty}^{\left[L^S_{i(j-1)}\Sigma\right]_{k,.}\mathbf{t}}\left(\int_{-\infty}^{[L^S_{i(j-1)}\Sigma]_{-k,.}\mathbf{t}} f([\mathbf{z}]_{-k}|z_k)d[\mathbf{z}]_{-k}\right)f(z_k)dz_k\right)\frac{\partial\left(\left[L^S_{i(j-1)}\Sigma\right]_{k,.}\mathbf{t}\right)}{\partial t_\ell}$$

$$=\sum_{k=1}^{j-1}\left[L^S_{i(j-1)}\Sigma\right]_{k,\ell}\Phi^{(j-2)}\left([L^S_{i(j-1)}\Sigma]_{-k,.}\mathbf{t};\tilde{\boldsymbol{\mu}}_{ij|k}\left(z_k=[L^S_{i(j-1)}\Sigma]_{k,.}\mathbf{t}\right),\tilde{\Sigma}_{ij|k}\right)$$
$$\times\,\phi\left([L^S_{i(j-1)}\Sigma]_{k,.}\mathbf{t};[\tilde{\boldsymbol{\mu}}_{ij}]_k,[\tilde{\Sigma}_{ij}]_{k,k}\right).$$

When $\mathbf{t}=\mathbf{0}$ we have

$$\frac{\partial}{\partial \mathbf{t}_\ell}\int_{-\infty}^{L^S_{i(j-1)}\Sigma\mathbf{t}} f(z_1,\cdots,z_{j-1})dz_1\cdots dz_{j-1}\,|_{\mathbf{t}=\mathbf{0}}$$
$$=\sum_{k=1}^{j-1}\left[L^S_{i(j-1)}\Sigma\right]_{k,\ell}\Phi^{(j-2)}\left(\mathbf{0};\tilde{\boldsymbol{\mu}}_{ij|k}(0),\tilde{\Sigma}_{ij|k}\right)\phi\left(0;[\tilde{\boldsymbol{\mu}}_{ij}]_k,[\tilde{\Sigma}_{ij}]_{k,k}\right).$$

Therefore,

$$\mathrm{E}(\mathbf{b}_i|S_i\geq j)$$
$$=\frac{\sum_{\ell=1}^q\sum_{k=1}^{j-1}[L^S_{i(j-1)}\Sigma]_{k,\ell}\Phi^{(j-2)}\left(\mathbf{0};\tilde{\boldsymbol{\mu}}_{ij|k}(0),\tilde{\Sigma}_{ij|k}\right)\phi\left(0;[\tilde{\boldsymbol{\mu}}_{ij}]_k,[\tilde{\Sigma}_{ij}]_{k,k}\right)\mathbf{e}_\ell}{\Phi^{(j-1)}(0;\tilde{\boldsymbol{\mu}}_{ij},\tilde{\Sigma}_{ij})},$$

where $\mathbf{e}_\ell$ is a unit vector whose components are zero, except the ℓth component which is one.

If $j=2$, the conditional expectation is simplified as,

$$\mathrm{E}(b_i|S_i>1)=\frac{\sum_{\ell=1}^q[L^S_{i1}\Sigma]_\ell\phi(0;-X_{S,i1}\boldsymbol{\alpha}^S,1+L^S_{i1}\Sigma(L^S_{i1})^{\mathrm{T}})\mathbf{e}_\ell}{\Phi^{(1)}(0;-X_{S,i1}\boldsymbol{\alpha}^S,1+L^S_{i1}\Sigma(L^S_{i1})^{\mathrm{T}})}.$$

3 Further results from the HERS analysis

Figure 1 presents both the unconditional and conditional longitudinal profiles for the HERS patients with different baseline viral loads. Based on the unconditional longitudinal profiles, patients with higher baseline viral load had much lower CD4 counts at baseline and steeper decline over the follow-up. As the partly conditional mean profiles take into account the survival differences over time and between baseline HIV viral load groups, the differences

between the conditional profiles from different baseline HIV viral load groups are reduced compared with those unconditional mean profiles. Therefore the interaction between time and baseline HIV viral load groups appears to be smaller because through the selection by survivals, the population remaining in the study is less heterogeneous even if the selection by dropout has been accounted for.

4 Simulation study

In this section, we perform a simulation study to evaluate the finite sample performance of the proposed joint model.

4.1 Design

The design of the simulation study is motivated by the HERS data in Section 4 of the main text. Specifically, we simulate the *complete* longitudinal measurements of CD4 count from the unconditional model as follows,

$$Y_{ij} = \mathbf{x}_{ij}^T \boldsymbol{\beta} + b_{i1} + b_{i2} j + \epsilon_{ij}, \tag{4}$$

where Y_{ij} is the square root of CD4 count at the jth ($j = 1, \ldots, 12$) visit and the covariate vector $\mathbf{x}_{ij}$ include the visit number j (time), indicator of antiretroviral therapy (ART) at baseline, and the interaction between time and ART at baseline. b_{i1} and b_{i2} are random intercept and slope, respectively; and they follow the multivariate normal distribution with mean zero and covariance $\varSigma$. The error term is $\epsilon_{ij} \overset{\text{iid}}{\sim} N(0, \sigma_\epsilon^2)$, The true values of $\boldsymbol{\beta}$, $\varSigma$ and σ_ϵ^2 are chosen based on the results from the Bayesian approach in Table 2 of the main text. We use the observed data for ART at baseline in the HERS data. Each simulated data set contains data from $N = 827$ patients and the complete longitudinal data for each patient contain 12 observations.

We simulate the true times for dropout and HIV-related death D_i and S_i based on the

following sub-models for dropout and HIV-related death,

$$\begin{aligned}
& Pr(D_i = j | D_i \geq j, \mathbf{x}_{D,ij}, b_{i1}, b_{i2}) \qquad (5) \\
= \; & 1 - \Phi\left\{\alpha_0^D + \alpha_1^D (j/12) + \alpha_2^D (j/12)^2 + \alpha_3^D ART_i + \gamma_1^D b_{i1} + \gamma_2^D b_{i2})\right\}, \\
& Pr(S_i = j | S_i \geq j, \mathbf{x}_{S,ij}, b_{i1}, b_{i2}) \\
= \; & 1 - \Phi\left\{\alpha_0^S + \alpha_1^S (j/12) + \alpha_2^S (j/12)^2 + \alpha_3^S ART_i + \gamma_1^S b_{i1} + \gamma_2^S b_{i2})\right\},
\end{aligned}$$

respectively, where $j = 1, \ldots, 12$ and ART_i is the indicator of ART at baseline. Then the observed dropout time D_i^* and δ_i^D, and the observed time for HIV-related death S_i^* and δ_i^S are determined in four scenarios described in Section 1 as follows. If $D_i > 12$ and $S_i > 12$, both D_i and S_i are censored at the 12th visit, i.e., $C_i = 12$, $D_i^* = 12$, $\delta_i^D = 0$, $S_i^* = 12$ and $\delta_i^S = 0$. If $D_i \leq 12 < S_i$ then $D_i^* = D_i$ and $\delta_i^D = 1$, $S_i^* = 12$ and $\delta_i^S = 0$. If $S_i < D_i$ and $S_i \leq 12$, we censor the dropout time at S_i such that $D_i^* = S_i$ and $\delta_i^D = 0$, $S_i^* = S_i$ and $\delta_i^S = 1$. If $D_i \leq S_i \leq 12$, then $D_i^* = D_i$ and $\delta_i^D = 1$, $S_i^* = S_i$ and $\delta_i^S = 1$. Finally we truncate the complete longitudinal data $\mathbf{Y}_i$ at the jth visit that $j = \min(D_i^*, S_i^*)$ to create the observed longitudinal data $\mathbf{Y}_i^o$.

Under the above settings, we simulate 200 datasets and fit the joint model with the correct specification as in (4) and (5). The estimation is performed using the Bayesian approach. We assign independent Normal priors $N(0, 100)$ to $\boldsymbol{\beta}$ and the parameter λ_{21} in Σ. For parameters in the dropout and death models, we assign weakly informative $N(0, 4)$ priors to $\boldsymbol{\alpha}^D$, γ_1^D,γ_2^D, $\boldsymbol{\alpha}^S$, γ_1^S,γ_2^S because these models are specified at the probit scale. For variance component parameters, we assign the prior $\sigma_\epsilon^2 \sim$ Inverse-Gamma$(0.001, 0.001)$ and $\sigma_k^2 \sim$ Inverse-Gamma$(0.01, 1)$ $(k = 1, 2)$ for Σ. We run two MCMC chains with diverse initial values and use a $1,000$-iteration burn-in period. The pooled posterior samples of size $4,000$ are used for model inference.

4.2 Results

For regression coefficients and variance component parameters in the longitudinal model (Table 1), the posterior mean estimates based on the proposed joint model had minimal

absolute biases and the 95% credible intervals had good coverage probabilities.

Due to the inherent correlations between $j/12$ and $(j/12)^2$, the estimation for their regression coefficients in the dropout and HIV-related death models is not stable with high correlations in their posterior samples. However, the corresponding regression functions $\alpha_0^D + \alpha_1^D(j/12) + \alpha_2^D(j/12)^2$ and $\alpha_0^S + \alpha_1^S(j/12) + \alpha_2^S(j/12)^2$ at $j = 1, \ldots, 12$ are estimated well (Table 2). The only exception is for $\alpha_0^S + \alpha_1^S(j/12) + \alpha_2^S(j/12)^2$ at early follow-up $j = 1, 2$ and later follow-up $j = 12$, which had lower than nominal coverage probabilities and slightly larger biases. This probably can be explained by the large curvature ($\alpha_0^S = 3.472$, $\alpha_1^S = -4.272$, $\alpha_2^S = 2.931$) in the true regression function for the HIV-related death model such that at the boundaries of the regression function (in the early and later follow-up) estimation is difficult. In addition, compared with the parameters in the dropout model, the parameter estimates in the HIV-related death model had larger empirical standard deviations, which is probably due to the high survival probabilities (e.g., $\alpha_0^S = 3.472$) in the design and the consequent lack of information from the data for estimation.

References

Arnold, B. C. (2009) Flexible univariate and multivariate models based on hidden truncation. *Journal of Statistical Planning and Inference*, **139**, 3741–3749.

Barrett, J., Diggle, P., Henderson, R. and Taylor-Robinson, D. (2015) Joint modelling of repeated measurements and time-to-event outcomes: flexible model specification and exact likelihood inference. *Journal of the Royal Statistical Society: Series B (Statistical Methodology)*, **77**, 131–148.

Flecher, C., Naveau, P. and Allard, D. (2009) Estimating the closed skew-normal distribution parameters using weighted moments. *Statistics and Probability Letters*, **79**, 1977–1984.

González-Farías, G., Domínguez-Molina, A. and Gupta, A. K. (2004) Additive properties of skew normal random vectors. *Journal of Statistical Planning and Inference*, **126**, 521–534.

Figure 1: Estimated longitudinal profiles (posterior mean) of CD4 count in the HERS data for four baseline HIV viral load groups (with 1 HIV symptom and art at baseline).

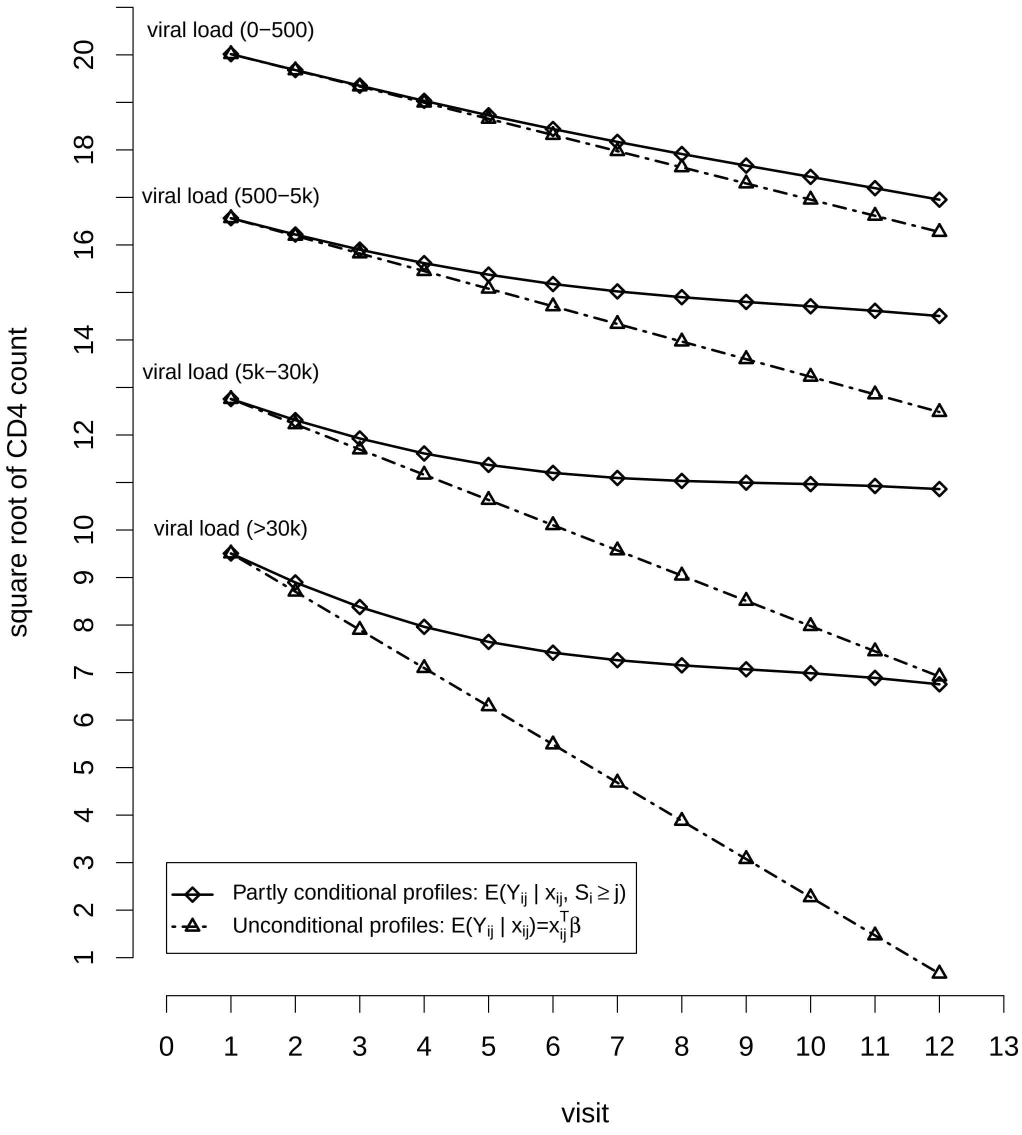

Table 1: Bias, empirical standard deviation (SD) for parameter estimates (posterior means) and coverage probability of the 95% credible intervals from the longitudinal part of the joint model (using the Bayesian approach) fitted to the 200 simulated datasets.

Parameter	True	Mean	Bias	Empirical SD	Coverage probability
**Longitudinal**					
Intercept	15.08	15.10	0.02	0.25	96.0
Time (visit)	-0.86	-0.88	-0.01	0.05	93.0
ART at baseline	-4.65	-4.58	0.07	0.43	96.5
Time*ART at baseline	0.11	0.11	0.00	0.08	93.5
σ_ϵ^2	7.30	7.30	-0.01	0.18	95.0
var(b_{i1})	29.12	29.17	0.05	1.72	96.5
var(b_{i2})	0.54	0.55	0.01	0.05	92.0
cov(b_{i1}, b_{i2})	-1.21	-1.21	0.00	0.25	95.5
corr(b_{i1}, b_{i2})	-0.31	-0.30	0.01	0.05	95.5

Table 2: Bias, empirical standard deviation (SD) for parameter estimates (posterior means) and coverage probability of the 95% credible intervals from the dropout and HIV-related death parts of the joint model (using the Bayesian approach) fitted to the 200 simulated datasets.

Parameter		True	Mean	Bias	Empirical SD	Coverage probability
**Dropout**						
$\alpha_0^D + \alpha_1^D(j/12) + \alpha_2^D(j/12)^2$:						
	$j = 1$	1.14	1.16	0.02	0.05	92.0
	$j = 2$	1.20	1.21	0.00	0.04	95.0
	$j = 3$	1.24	1.23	-0.01	0.04	94.5
	$j = 4$	1.26	1.24	-0.02	0.04	94.0
	$j = 5$	1.25	1.22	-0.02	0.04	93.5
	$j = 6$	1.21	1.19	-0.03	0.04	92.0
	$j = 7$	1.16	1.13	-0.03	0.04	91.0
	$j = 8$	1.08	1.05	-0.02	0.04	93.0
	$j = 9$	0.97	0.95	-0.02	0.05	94.5
	$j = 10$	0.84	0.83	-0.01	0.05	95.5
	$j = 11$	0.69	0.69	0.00	0.07	94.0
	$j = 12$	0.51	0.52	0.01	0.09	94.0
ART at baseline		-0.04	-0.03	0.01	0.05	96.0
γ_1^D		0.03	0.03	0.00	0.01	93.0
γ_2^D		0.44	0.46	0.01	0.06	94.0
**HIV-related death**						
$\alpha_0^S + \alpha_1^S(j/12) + \alpha_2^S(j/12)^2$:						
	$j = 1$	3.14	3.00	-0.14	0.15	81.5
	$j = 2$	2.84	2.75	-0.09	0.13	85.5
	$j = 3$	2.59	2.53	-0.06	0.11	90.0
	$j = 4$	2.37	2.35	-0.03	0.10	92.5
	$j = 5$	2.20	2.20	-0.01	0.09	95.5
	$j = 6$	2.07	2.08	0.01	0.08	94.5
	$j = 7$	1.98	1.99	0.01	0.08	94.5
	$j = 8$	1.93	1.94	0.01	0.07	94.5
	$j = 9$	1.92	1.92	0.00	0.07	95.5
	$j = 10$	1.95	1.93	-0.02	0.07	95.0
	$j = 11$	2.02	1.97	-0.05	0.08	91.5
	$j = 12$	2.13	2.05	-0.08	0.10	87.0
ART at baseline		-0.52	-0.48	0.04	0.09	95.0
γ_1^S		0.13	0.12	0.00	0.01	92.0
γ_2^S		1.19	1.14	-0.05	0.13	90.5
